# Theoretical Assessment of the Ligand/Metal/Quadruplex Recognition in the Non-Canonical Nucleic Acids Structures

**DOI:** 10.3390/molecules28166109

**Published:** 2023-08-17

**Authors:** Nikoleta Kircheva, Stefan Dobrev, Vladislava Petkova, Snezhana Bakalova, Jose Kaneti, Silvia Angelova

**Affiliations:** 1 Institute of Optical Materials and Technologies “Acad. J. Malinowski”, Bulgarian Academy of Sciences, 1113 Sofia, Bulgaria; nkircheva@iomt.bas.bg (N.K.); sdobrev@iomt.bas.bg (S.D.); vpetkova@iomt.bas.bg (V.P.); 2 Institute of Organic Chemistry with Centre of Phytochemistry, Bulgarian Academy of Sciences, 1113 Sofia, Bulgaria; snezhana.bakalova@orgchm.bas.bg (S.B.); jose.kaneti@orgchm.bas.bg (J.K.)

**Keywords:** quadruplex, guanine, G-tetrad, potassium, 4-(3,4-dihydroisoquinolin-2-yl)-2-(quinolin-2-yl)quinazoline

## Abstract

Quadruplexes (GQs), peculiar DNA/RNA motifs concentrated in specific genomic regions, play a vital role in biological processes including telomere stability and, hence, represent promising targets for anticancer therapy. GQs are formed by folding guanine-rich sequences into square planar G-tetrads which stack onto one another. Metal cations, most often potassium, further stabilize the architecture by coordinating the lone electron pairs of the O atoms. The presence of additional nucleic acid bases, however, has been recently observed experimentally and contributes substantially to the structural heterogeneity of quadruplexes. Therefore, it is of paramount significance to understand the factors governing the underlying complex processes in these structures. The current study employs DFT calculations to model the interactions between metal cations (K^+^, Na^+^, Sr^2+^) and diverse tetrads composed of a guanine layer in combination with a guanine (G)-, adenine (A)-, cytosine (C)-, thymine (T)-, or uracil (U)-based tetrad layer. Moreover, the addition of 4-(3,4-dihydroisoquinolin-2-yl)-2-(quinolin-2-yl)quinazoline to the modeled quadruplexes as a possible mechanism of its well-exerted antitumor effect is assessed. The calculations imply that the metal cation competition and ligand complexation are influenced by the balance between electronic and implicit/explicit solvation effects, the composition of the tetrad layers, as well as by the solvent exposure to the surrounding environment expressed in terms of different dielectric constant values. The provided results significantly enhance our understanding of quadruplex diversity, ligand recognition, and the underlying mechanisms of stabilization at an atomic level.

## 1. Introduction

Ever since the fundamental suggestion of the DNA double-stranded right-handed helix structure by Watson and Crick [1], the quest for unraveling its enigmatic and fascinating composition has tempted many researchers from diverse scientific fields. Since then, however, numerous peculiar DNA/RNA motifs have been discovered, with one such example posing the so-called quadruplexes. Notably, these motifs are enriched in certain regions rather than being randomly distributed throughout the genome. This strictly corresponds to the key part they play in the regulation of various biological processes, e.g., mitochondrial transcription, telomere biogenesis and homeostasis, mRNA translocation, transcription, replication, and chromatin remodeling, just to name a few [2,3,4,5,6,7]. In the context of their diverse functions, especially in telomere regulation and homeostasis, quadruplexes have been recognized as a plausible drug target in anticancer therapy against most tumor formations [8,9,10,11]. Therefore, it is of utmost interest and importance to understand the underlying factors that govern the intra- and intermolecular interactions observed in their complex architecture.

**Figure 1:**
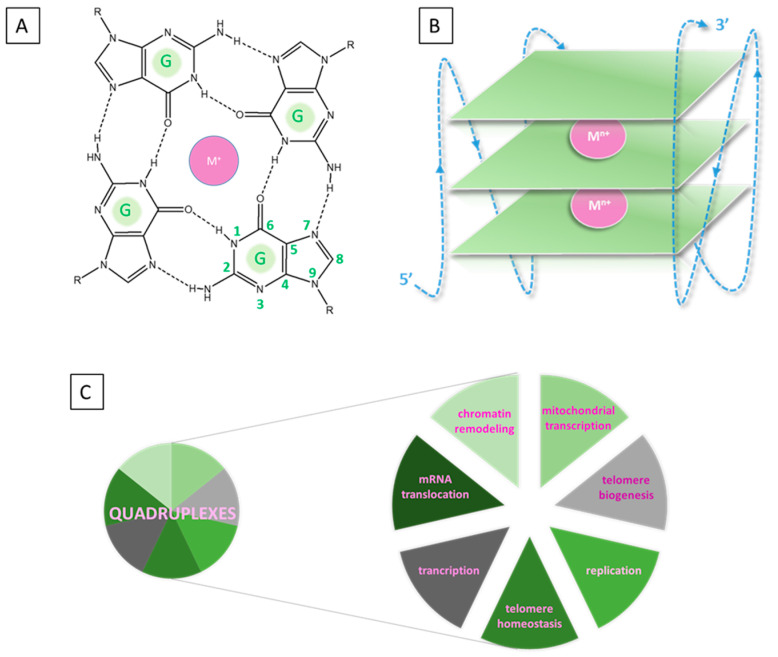
Guanine structures constructed via HG base pairs from guanine: 2D model of a G-quartet (**A**) and schematic structure of a quadruplex (**B**); function of quadruplexes (**C**).

Quadruplexes are most commonly formed by folding guanine-rich nucleic acid sequences in both RNA and DNA in the arrangement of square planar compositions due to the presence of complementing hydrogen bonds (N1-O6)(N2-N7) on their Hoogsteen (HG) and Watson–Crick (WC) faces (Figure 1).

The arising G-quartets (GQs) or G-tetrads stack onto one another. It is further stabilized by the addition of a metal cation, usually potassium (K^+^) or, much less frequently, sodium (Na^+^), although other (mostly alien) metal species have additionally been observed to have an effect [12,13,14,15,16]. The metal cofactor is a highly required component, which stabilizes the intricate architecture through coordination with the oxygen lone pairs in the central channel of the quadruplex. Depending on the chemical characteristics of the metal cation (ionic radius, charge, coordination preferences) and the structure of the GQs, the M^n+^ is positioned either within the approximate tetrad plane or between two planes. In the nucleic sequence, the intervening bases between the G-tetrads build the so-called loops whose function is to link the stacked G-quartets [17,18,19]. Hence, the typical composition of a quadruplex consists of three main constituents: (1) layers of rigid coplanar G-tetrads, (2) flexible linking loops, and (3) a central alkali metal cation, which is present in almost all structures. This structural polymorphism results in great diversity in loop and tetrad arrangements, capping structures, and strand orientation. Furthermore, their various conformations (parallel, antiparallel, hybrid-I and II) are not constant but could undergo conversion when the environmental conditions change [20]. With the development of experimental techniques like nuclear magnetic resonance (NMR), circular dichroism (CD), and gel electrophoresis, some interesting nontypical tetrads have been recently observed, namely A-/C-/U- and T-tetrads [21,22,23,24,25,26,27,28,29,30]. These are commonly stacked over the adjacent G-quartets and add significantly to the structural diversity and recognition especially in telomeres.

The unceasing search for novel anticancer therapeutics that exert not only selective effect but possess a different mechanism of action that could circumvent the increasing tumor resistance against conventional drugs has led to the recognition of the crescent-shaped planar heterocycles targeting the quadruplex architecture [31,32,33,34,35]. Thus, the resulting stabilization and/or induction of quadruplexes due to interaction with the ligand may lead to (1) maintaining telomere problems (decreasing telomerase activity and increasing telomere stability); (2) reducing oncogene expression by inhibiting transcription and translation; (3) increasing genome instability; hence, inducing apoptosis (cell death). In this regard, immense effort has been made, but still, no applicable drug has reached the market up to date. Herewith, we continue our previous work [36,37] on studying the possible complexation between heterocyclic molecules (quinazoline derivatives) and G-quadruplexes. Our main goal in the current study is, however, to decipher the principal factors that govern the recognition between three metal cations (K^+^, Na^+^, and Sr^2+^) and the intriguing non-canonical quadruplexes by modeling their interaction with combinations of tetrads. Furthermore, we evaluate the possible stacking of the most active (lowest IC_50_) 2-quinolinyl-quinazoline derivative reported previously in [37] to the modeled tetrads. The results are obtained by utilizing a well-studied and validated DFT methodology that has proven extensively reliable when the system under study contains competing metal cofactors which bind to biological or biomimetic molecules through mostly electrostatic interactions [38,39,40,41,42]. Thus, we aim to contribute to the understanding of the underlying processes in quadruplex stabilization and its further complexation to drug molecules.

## 2. Results

### 2.1. Modeled Reactions

The main objective of the current study is to outline the specific effects that control the metal recognition in nucleic tetrads, as well as the influence that these conditions may have upon the addition of an outer ligand with potential anticancer activity. In order to achieve this goal, we modeled the following reactions of competition and addition:

Na(H_2_O)_6_^Ⴈ+^ + K@GQ^Ⴈ+^ → Na@GQ^Ⴈ+^ + K(H_2_O)_6_^Ⴈ+^ (R1)

Sr(H_2_O)_8_^Ⴈ2+^ + K@GQ^Ⴈ+^ → Sr@GQ^Ⴈ2+^ + K(H_2_O)_6_^Ⴈ+^ + 2H_2_O (R2)

L + K@GQ → L@K@GQ^Ⴈ+^ (R3)

where Na(H_2_O)_6_^Ⴈ+^, K(H_2_O)_6_^Ⴈ+^, and Sr(H_2_O)_8_^Ⴈ2+^ represent the corresponding hydrated species of the studied metal cations; M@GQ^Ⴈn+^ stands for the metal-containing structure of two stacked tetrads; the considered ligand L is 4-(3,4-dihydroisoquinolin-2-yl)-2-(quinolin-2-yl)quinazoline in its “concave” form; and the L@K@GQ^Ⴈ+^ complex depicts the resulting adduct between the ligand and the initial metal-containing quadruplex structure yielded in Reaction (R3). This particular ligand was chosen as it was previously demonstrated to express the most potent cytotoxicity effect on A375 melanoma cells (81% and 91% reduction at the low and higher concentrations, respectively) according to Ref. [37]. Four different rotamers of the ligand were furthermore studied, and the most stable one was considered as the structure of choice in the current calculations (Figure S1).

**Figure 2:**
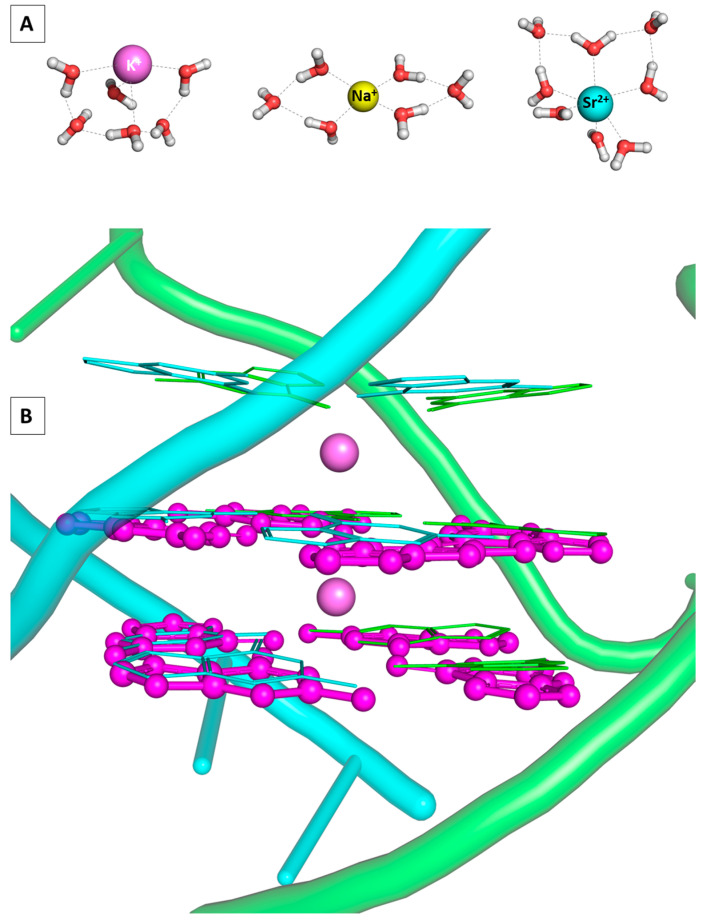
Structures of the fully optimized Na(H_2_O)_6_^Ⴈ+^, K(H_2_O)_6_^Ⴈ+^, and Sr(H_2_O)_8_^Ⴈ2+^ constructs at the wB97XD/6-31G(d,p) level used in the current study (**A**); juxtaposed PDB deposited (3IBK) and GG quadruplex models, fully optimized in the gas phase, colored in light blue and purple, correspondingly (**B**).

The number of water molecules surrounding the metal species under study varies greatly (between 5 and 7 for Na^+^, from 6 to 8 for K^+^, and from 5 to 9 for Sr^2+^) depending on the environment and experimental/theoretical approach used [43,44,45,46]. For the purposes of the current study and based on our previous work with these metal species, the Na(H_2_O)_6_^Ⴈ+^, K(H_2_O)_6_^Ⴈ+^, and Sr(H_2_O)_8_^Ⴈ2+^ constructs were taken into account when modeling the envisioned reactions [41,47]. These substantially correspond to experimental and theoretical data provided and reproduce structural characteristics such as bond lengths and valence angles: the 4 + 2 composition of the sodium and potassium-hydrated species as seen in [43,48,49] along with the 6 + 2 preference of the strontium cation [50]. These particular compositions are consistent with structures in which the first coordination sphere consists of four/six water molecules for the mono-and divalent cations, respectively, whereas the remaining two water molecules migrate to the second one, distanced at more than 4 Å away from the metal cation but bound to the structure by hydrogen bonds. These were found to be the most energetically favorable geometries for the studied ions among others taken into consideration, as presented in Figure S1. Furthermore, the quadruplex architectures are composed by two layers, one of which is a G-quartet, whereas the other one is built by four guanines, adenines, cytosines, thymines, or uracils, henceforth referred to as GG, AG, CG, TG, and UG in the text. Their initial geometries are taken from the data deposited in the Protein Data Bank structures—PDB Entries 3IBK [51], 1EVN [22], 1EVO [24], 1EMQ [23], and 6GE1 [52], correspondingly, followed by full geometry optimization and vibrational frequency analysis (see Materials and Methods). Note that all chosen initial structures accept parallel geometry. The architectures of the stacked tetrads are considered as two layers of four building nucleic bases each, held by the arising Hoogsteen interactions and the complementing metal cation complexation due to the computational restriction of the DFT methodology concerning the size of the studied systems. Thus, the linking loops as well as the phosphate moieties at the side chain of the quadruplexes are omitted. Note, however, that this simplification does not significantly alter the overall architecture, as seen in Figure 2, where the PDB deposited and the optimized K@GG^Ⴈ+^ are juxtaposed.

### 2.2. Replacement of K^+^ with Na^+^

**Figure 3:**
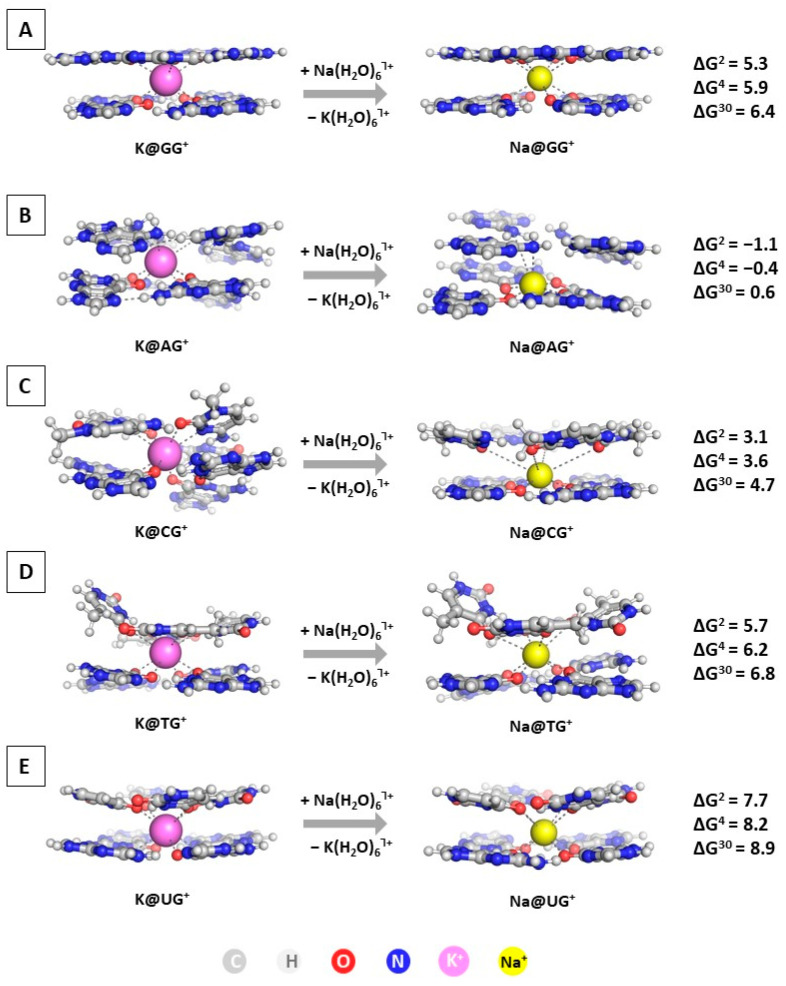
Gibbs energies of substitution of K^+^ with Na^+^ (in kcal mol^−1^) in three environments of different polarity for wB97XD/6-31+G(d,p)//wB97XD/6-31G(d,p) optimized M@GQ^Ⴈ+^ complexes: M@GG^Ⴈ+^ (**A**); M@AG^Ⴈ+^ (**B**); M@CG^Ⴈ+^ (**C**); M@TG^Ⴈ+^ (**D**); M@UG^Ⴈ+^ (**E**). The color scheme is further applied in all presented figures.

The first step in the current study is modeling the substitution of K^+^ with Na^+^ in the GQ structures. The optimized geometries of the M@GQ^Ⴈ+^ complexes, along with the obtained results at the wB97XD/6-31+G(d,p)//wB97XD/6-31G(d,p) level of theory in accordance to Reaction (R1), are presented in Figure 3.

The calculations reveal that the most common and widely distributed G-quadruplex built by G-stacked tetrads is protected against alien attack: the ∆G^ε^ values stay on positive ground ranging from 5.3 to 6.4 kcal mol^−1^. This result corresponds well to experimental data claiming the inability of the sodium cation to compete with the native potassium one in these structures [13,14,16,19,35]. Moreover, the obtained positive changes in the ∆G^ε^ values for the substitution of K^+^ with Na^+^ in the CG, TG, and UG compositions add further evidence to this concept. Interestingly, the AG structure appears vulnerable to a sodium attack, as the ∆G^2/4/30^ values are −1.1/−0.4/0.6. Calculations at the lower wB97XD/6-31G(d,p)//wB97XD/6-31G(d,p) level show a similar trend, although with lower ∆G numbers in absolute value (compare results in Table S1). Note that Na^+^ takes the position in the plane of the G-tetrad in the Na@AG^Ⴈ+^ and Na@CG^Ⴈ+^ complexes (at about 0.2 Å closer to the guanine quadruplex), whereas it stands between two layers in all other Na@GQ^Ⴈ+^. The substitution of the native potassium with the alien sodium ion in these constructs leads either to energy gain (in the case of an AG tetrad) or yields the lowest positive ∆G^ε^ values. Hence, the former octahedral configuration appears more stable for the smaller sodium cation as opposed to its latter bipyramidal geometry, as it provides greater interaction with the bases of the layer. Both of these compositions are common, in addition to the unsymmetrical geometry of Na^+^ [12,19]; however, the Na^+^/K^+^ rivalry is most probably dominated by the intracellular concentration of the cations (5–15 mM Na^+^ and 140 mM K^+^ [53]) followed by the position of the sodium ion.

### 2.3. Replacement of K^+^ with Sr^2+^

**Figure 4:**
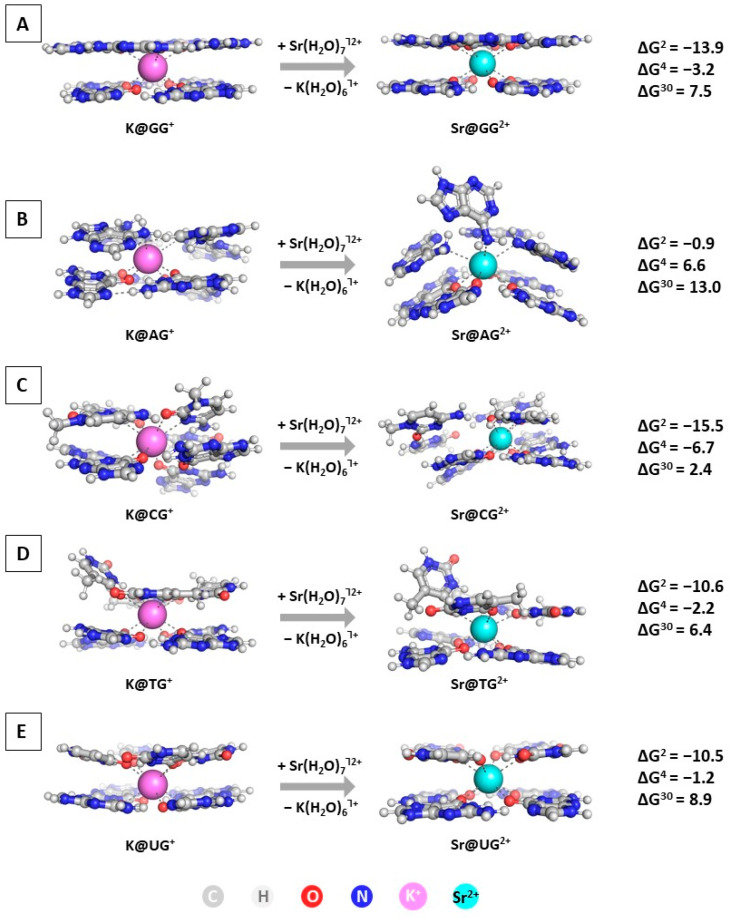
Gibbs energies of substitution of K^+^ with Sr^2+^ (in kcal mol^−1^) in three environments of different polarity for wB97XD/6-31+G(d,p)//wB97XD/6-31G(d,p) optimized M@GQ^Ⴈ+/2+^ complexes: M@GG^Ⴈ+/2+^ (**A**); M@AG^Ⴈ+/2+^ (**B**); M@CG^Ⴈ+/2+^ (**C**); M@TG^Ⴈ+/2+^ (**D**); M@UG^Ⴈ+/2+^ (**E**).The applied color scheme has been previously presented in Figure 3.

The next step in the current study is modeling the substitution of K^+^ with Sr^2+^ in the GQ structures. The optimized geometries of the M@GQ^Ⴈ+/2+^ complexes, along with the obtained results at the wB97XD/6-31+G(d,p)//wB97XD/6-31G(d,p) level of theory in accordance to Reaction (R2), are presented in Figure 4.

The performed calculations at the two different levels (Figure 4 and Table S1) provide interesting results regarding the importance of diverse factors controlling the native/abiogenic ion competition. The alien strontium cation effectively replaces the native K^+^ in all studied constructs in a nonpolar environment characterized by a dielectric constant ε = 2, as all of the obtained ∆G^2^ values are strongly negative: ranging from −0.9 kcal mol^−1^ for the substitution in the M@AG^Ⴈ+/2+^ complex to −15.5 kcal mol^−1^ in the M@CG^Ⴈ+/2+^ structure. This outcome should be attributed to the greater complexation ability of Sr^2+^ due to its higher charge in combination with the plausible ionic radius resembling that of the biogenic potassium (1.32 Å vs. 1.38 Å for Sr^2+^/K^+^, respectively [54]). Nevertheless, with increasing the dielectric constant of the medium, corresponding to greater solvent exposure, the substitution of K^+^ with Sr^2+^ becomes thermodynamically unfavorable in all modeled reactions (positive ∆G^4/30^ values), with the M@CG^Ⴈ+/2+^ structure posing the only exception where the reaction yields a positive ∆G^4^. The explanation lies in the enhanced desolvation penalty for the strontium cation in the more polar environment. Noteworthy, the polarity of the medium plays a significant role, as with increasing the dielectric constant the energy gain decreases substantially. Interestingly, these results correlate to contradicting experimental data, as some authors provide clear evidence of the ability of strontium to substitute the native potassium [12], whereas others claim the opposite trend [13]. Our computational approach delineates the importance of the medium surrounding these DNA/RNA motifs, as well as the structure of the building tetrad that undoubtedly affects the outcome of the Sr^2+^/K^+^ rivalry.

### 2.4. Ligand Complexation by the Studied K@GQ^Ⴈ+^ Structures

**Figure 5:**
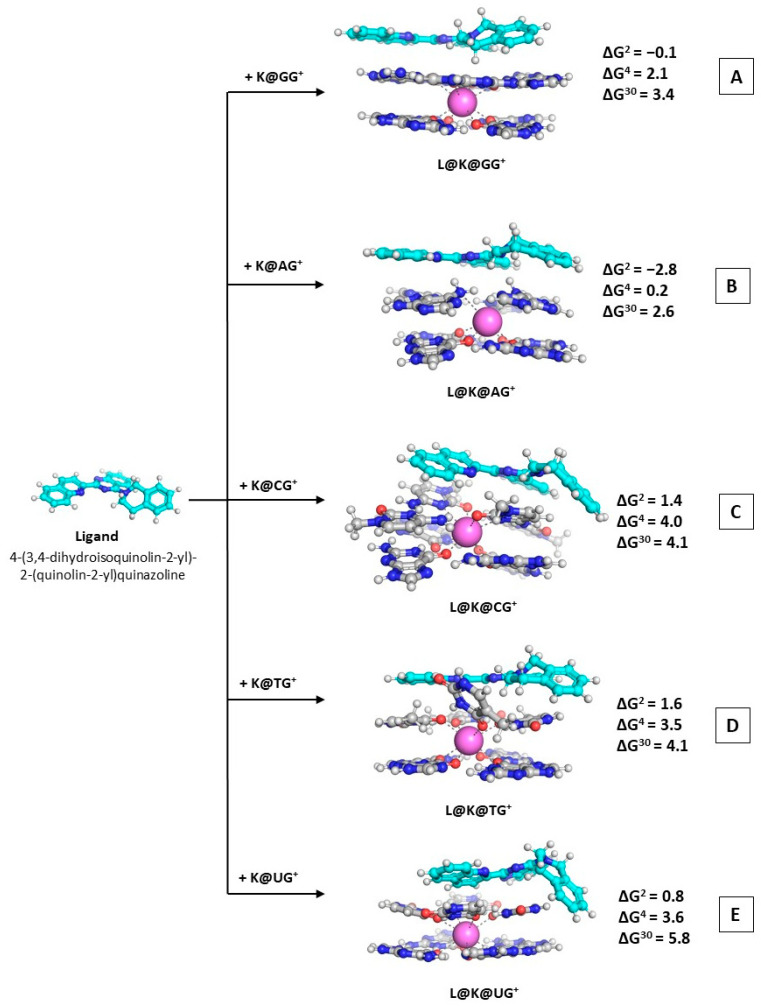
Gibbs energies of formation (in kcal mol^−1^) in three environments of different polarity for wB97XD/6-31+G(d,p)//wB97XD/6-31G(d,p)optimized L@K@GQ ^Ⴈ+^ ternary complexes: L@M@GG^Ⴈ+^ (**A**); L@M@AG^Ⴈ+^ (**B**); L@M@CG^Ⴈ+^ (**C**); L@M@TG^Ⴈ+^ (**D**); L@M@UG^Ⴈ+^ (**E**). The applied color scheme has been previously presented in Figure 3.

As a continuation of our previous work [36,37], we further modeled the reaction of complexation between the most potent heterocyclic molecule exhibiting the greatest cytotoxic effect and all five types of K@GQ^Ⴈ+^ complexes in order to assess its ability to bind them. The most stable rotamer of ligand L was used in modeling the complexes with K@GQ^Ⴈ+^ (the relative stabilities of possible rotamers of L are shown in (Figure S1, Supplementary Materials). The optimized structures of the ternary complexes denoted as L@K@GQ^Ⴈ+^ are depicted in Figure 5. The obtained ∆G^ε^ values regarding Reaction (R3) are provided as well.

The calculated Gibbs energies in the nonpolar environment stay on negative ground or close to zero, indicating a thermodynamically favorable or probable reaction of formation. The ∆G^ε^ values vary between −2.8 and 1.6 kcal mol^−1^ at ε = 2, between 0.2 and 4.0 kcal mol^−1^ at ε = 4, and between 2.6 and 5.8 kcal mol^−1^ at ε = 30. This fluctuation further reveals the significant role of the surrounding medium (the reactions of formation of the L@K@GG^Ⴈ+^ and L@K@AG^Ⴈ+^ complexes appear thermodynamically favorable (exergonic) in the deeply buried nonpolar environment (∆G^2^ = −0.1/−2.8 kcal mol^−1^, respectively) whereas the ∆G^30^ values become positive (3.4/2.6 kcal mol^−1^, correspondingly) but still stay close to zero. Moreover, the obtained result through the currently applied computational protocol of ∆G^2^ = −2.4/−0.1 kcal mol^−1^ (higher level vs. lower level of theory) for the formation of L@K@GG^Ⴈ+^ provides evidence of the expressed hypothesis that the ligand’s mechanism of action is due to stacking to the quadruplex architecture, hence the previously reported low IC_50_ value of 1.43 × 10^−3^ M. Notably, the rest of the GQ-stacked tetrads further appear prone to positive interaction with the ligand under study, evidenced by the negative ∆G^2^ values at the wB97XD/6-31G(d,p)//wB97XD/6-31G(d,p), and, all the more, the K@AG^Ⴈ+^ motif with negative values even at the higher wB97XD/6-31+G(d,p)//wB97XD/6-31G(d,p) level of theory. This outcome unambiguously proves that 4-(3,4-dihydroisoquinolin-2-yl)-2-(quinolin-2-yl)quinazoline is indeed a potent anticancer drug exerting its effect (at least partially) by successfully binding the quadruplex architectures in telomeres.

## 3. Discussion

### 3.1. Effect of the Tetrad Nature

G-stacked tetrads constitute most commonly quadruplexes in telomeres playing a role of high significance in cell apoptosis. Recent studies, however, have revealed even more peculiar motifs, where in addition to the G-layers, tetrads composed of other nucleic bases, both of a purine and pyrimidine nature, are observed. The current study delineates the importance of the present bases for the outcome in metal competition as well as ligand addition to the structures. The absence of carbonyl groups in the adenine tetrad, as well as the presence of only one per base in the cytidine quadruplex, predispose the smaller sodium cation to migration, thus occupying the position in the plane of the G-quartet in an octahedral geometry. This results in a positive outcome (negative ∆G^ε^ values) of the competition with K^+^ in the M@AG^Ⴈ+^ construct, which is the most commonly present metal cation stabilizing the quadruplex architecture. Notably, the presence of more carbonyl groups in the remaining studied bases inclines the metal cations to accommodate in the position between two layers, which negatively affects the outcome in the Na^+^ → K^+^ substitution (positive ∆G^ε^ values). This is especially underlined in the case of the Na@UG^Ⴈ+^ complex, where the attacking ion is unable to substitute the native potassium, evidenced by the highest yielded ∆G^ε^ values. The same conclusion can be drawn regarding the Sr^2+^/K^+^ competition for binding the AG -composition, where ∆G^2^ equals −0.9 kcal mol^−1^. Although the result stays below zero, this is the smallest in absolute value yielded for the substitution of K^+^ with Sr^2+^ at ε = 2. Therefore, the optimization of the structures under study points out that the tetrad nature affects mostly the position of the smaller Na^+^, while the bulkier K^+^ and Sr^2+^ experience only loss of interaction but remain between two layers due to their ionic radii. Furthermore, the arising interaction with the NH_2_ groups favors the addition of 4-(3,4-dihydroisoquinolin-2-yl)-2-(quinolin-2-yl)quinazoline to the K@AG^Ⴈ+^ complex to the greatest extent, indicated by the lowest ∆G^ε^ values. Still, it should be noted that the studied ligand successfully binds to the most abundant quadruplex construct, the GG tetrad, displaying a potential for application in antitumor therapy aimed at interaction with quadruplexes in telomeres. Overall, the K@AG^Ⴈ+^ compositions appear the most vulnerable towards an outer attack, either by a competing metal cation or by an external ligand binding to the complex, evidenced by the performed calculations.

### 3.2. Effect of the Metal Cation Properties

The conducted study reveals the significance of the metal cation properties for elucidating the outcome of the Na^+^/K^+^ and Sr^2+^/K^+^ rivalry for binding diverse G-quadruplex architectures by utilizing a well-defined and previously applied DFT methodology. This particular approach outlines the most important factors: the ionic radius, charge, and de/hydration energy of the metal cation. Notably, the obtained results fall strictly in line with previously observed experimental trends but draw a clearer picture at an atomic level. The data demonstrate that metal cations characterized by an ionic radius of around 1.35 Å bind most effectively the lone electron pairs of the O/N atoms of the tetrad bases when positioned between two layers in a bipyramidal geometry. Furthermore, the charge of the cation should also be taken into account as it corresponds to an increased ion–dipole interaction demonstrated by a Hirshfeld analysis [55]: 0.89 e^−^/0.77 e^−^ vs. 1.98 e^−^ for the monocationic K^+^/Na^+^, respectively, vs. the dicationic Sr^2+^ in the M@GG complexes. The analysis draws an analogous picture in all other compositions, as the charge transferred from the ligands to the metal cation varies between 0.81 and 0.86 e^−^ for K^+^, 0.71 and 0.75 e^−^ for Na^+^, and 1.93 and 1.98 e^−^ for Sr^2+^. The data clearly show the increased interaction between the potassium cation in comparison with the sodium one as well as the greater ion–dipole interaction with strontium, attributed to its higher charge. Nonetheless, the desolvation penalty of the M^n+^ attenuates the free energy gain, especially in the polar medium, thus settling the outcome of the metal cation rivalry. Consequently, the native potassium cation appears suitable for stabilizing the ion channel in the quadruplex architecture, but its extremely high cellular concentration should further be acknowledged. An even clearer picture regarding the significance of the nature of the metal cation in quadruplexes could be drawn in a future study focusing on the competition between K^+^ and a whole series of metal cations including trivalent ones, when the ionic polarity index presented in Ref. [56] could be applied.

### 3.3. Effect of the Dielectric Constant of the Medium

The results regarding ligand/metal/quadruplex recognition presented in the current study underline the considerable importance of the surrounding environment when modeling interactions in biological systems (inter/intracellular space, cytoplasm, nucleus, proteins). Firstly, the dielectric constant of the medium strongly influences the outcome of the metal competition at both levels of theory (Table S1). The interplay between the energy loss due to the desolvation of the incoming metal species and the energy gain from the solvation of the M^n+^ products results in effective competition of the alien strontium cation against the native potassium in an environment resembling the gas phase (negative ∆G^2^ in all cases and negative ∆G^4^ values in most of the studied tetrads for the substitution of K^+^ with Sr^2+^). Yet, this trend is reversed in the more polar surroundings, which substantially amplify the ion–dipole interaction in the Sr(H_2_O)_8_^Ⴈ2+^ construct. An analogous conclusion can be drawn regarding the Na^+^/K^+^ rivalry where the de/solvation energy of the much smaller sodium strongly surpasses that of the bulkier potassium. Moreover, the addition of the ligand to the K@GQ^Ⴈ+^ complexes is affected by the change in the dielectric constant of the medium, especially in the cases of the GG and AG architectures, where the change of the polarity of the environment reverses the sign of the Gibbs energy, thus indicating a thermodynamically unfavorable reaction under these conditions. Still, the calculations imply a plausible recognition between some K@GQ^Ⴈ+^ structures and the incoming ligand, especially in the less polar medium characterized by a dielectric constant of two.

### 3.4. Effect of the Ligand Addition

The previously reported cytotoxic analysis [37] provided evidence for the anticancer activity of the 4-(3,4-dihydroisoquinolin-2-yl)-2-(quinolin-2-yl)quinazoline in addition to theoretical assessment of its affinity to the quadruplex guanine tetrads. The computational protocol utilized herewith further assessed the change in the Gibbs energy in different media, thus providing negative ∆G^2^ value for the formation of L@K@GG^Ⴈ+^ that corresponds well to the experimentally observed low IC_50_ values, suggesting a probable mechanism of antitumor activity. Moreover, we demonstrate the ability of the ligand to bind effectively not only the GG-quartets, but also the AG-type non-canonical motif observed in telomeres, as the calculations stay firmly on negative ground: −2.8 kcal mol^−1^ in the nonpolar environment. The computed results differ with the change of the dielectric constant of the medium; hence, the values fluctuate between 0.2 and 4.0 kcal mol^−1^ when ε equals 4, and between 2.6 and 5.8 kcal mol^−1^ in the solvent-exposed environment. The calculations further imply that the tetrad most prone to ligand addition is the adenine-based quadruplex with ∆G^2/4/30^ values = −2.8/0.2/2.6 kcal mol^−1^, correspondingly, due to the arising interaction between the NH_2_ groups from the A bases and the N atoms in the heterocyclic rings in the ligand. This outcome provides strong evidence of the susceptibility of the non-canonical motifs to complexations to the potential drug molecule under study.

## 4. Materials and Methods

The DFT calculations were performed using the Gaussian 09 suite of programs [57]. The hybrid long-range and dispersion-corrected wB97XD functional [58,59] basis set, in conjunction with the 6-31G(d,p) basis set and the pseudopotential SDD [60] for the heavier Sr^2+^ in all cases, was employed for the full geometry optimization of the modeled structures, followed by vibrational frequency analysis where no negative values were observed. This outcome indicates a local minimum of the potential energy surface necessary for obtaining the electronic energy, E_el_, the thermal energy, E_th_, including the non-scaled zero-point energy, and entropy, S. They were further utilized in the calculation of the Gibbs energy value, ∆G^1^, in the gas phase at room temperature T = 298 K and atmospheric pressure 1 atm according to Equation (1):

∆G^1^ = ∆E_el_ + ∆E_th_ − T∆S (1)

where ΔE_el_, ΔE_th_, and ΔS represent the corresponding differences between the products and the reactants in consistency with Reactions R1 to R3. The change in the number of moles ∆n during the reaction is also accounted for in ∆G^1^ (∆nRT ≈ P∆V). Further assessment of the effect of the surrounding medium was obtained through additional SMD calculations [61]. Three different media characterized by a dielectric constant ε = 2, 4, and 30 were chosen, corresponding to a deeply buried, partially solvent-exposed, and solvent-accessible environment, respectively. “Generic” solvent option was used with Eps (2/4/30) and EpsInf (2) parameters specified. Single-point calculations for each of the optimized structures were performed and used for the evaluation of the solvation energies, ΔG_solv_^ε^, defined as the difference between the condensed-phase and the gas-phase energies of the respective constructs. These were employed for obtaining the energies for the substitution of K^+^ with Na^+^/Sr^2+^ and ligand addition to the already formed K@GQ^Ⴈ+^ structures in accordance with the equation:

∆G^ε^ = ∆G^1^ + ∆∆G_solv_^ε^ (2)

where

∆∆G_solv_^ε^ = ∆G_solv_^ε^ (products) − ∆G_solv_^ε^ (reactants) (3)

A positive ∆G_solv_^ε^ value characterizes a thermodynamically unfavorable reaction, whereas a negative ∆G_solv_^ε^ suggests a favorable one. In terms of selectivity, this outcome corresponds to a potassium-selective construct, while the opposite distinguishes a preference toward the foreign intruder and the addition of the outer ligand. In order to more adequately assess the underlying factors, additional single-point calculations with the triple-zeta 6-31+G(d,p) basis set for all optimized structures in the gas phase, as well as in different media, were employed. The obtained results are denoted as calculated at the wB97XD/6-31+G(d,p)//wB97XD/6-31G(d,p) level of theory. Further comparison between the two utilized schemes of computational methodologies is provided in Table S1 (Supplementary Materials), where additional presentation of the terms ∆H (accounting for ∆E_el_, ∆E_th_, and the change of the number of molecules at the two sides of the chemical equation) and T∆S is provided. This particular combination was chosen as it yielded positive ∆G^2^ value for the substitution of K^+^ with Na^+^ in the GG structure, indicating the inability of sodium to substitute the native potassium, falling in line with various experimental results observed in the literature. Note, however, that the aim of the performed calculations is not to reproduce specific values of ∆G_solv_^ε^ but, rather, to provide reliable trends that outline thermodynamic descriptors affecting the metal and ligand selectivity in nucleic quadruplexes. The PyMol molecular graphics system [62] is implemented for the visualization of the obtained results.

## 5. Conclusions

Guanine-rich motifs in telomeres have recently attracted the attention of scientists, not only due to their peculiarity, but also by their role in cells’ apoptosis, turning them into a plausible target in antitumor therapy. By utilizing the powerful tools of DFT computations, the current study adds to our understanding of the diversity of effects that play a role in defining the complex structure in quadruplexes. Overall, tetrad nature is of crucial importance for the modeled ligand/metal/quadruplex recognition. Apparently, the main function has been bestowed upon guanine as a leading base forming a great percentage of the quadruplex architectures by no chance, since it provides solid structural integrity evidenced by the performed computations: the layered composition is not altered either by the addition of metal cations or by the complexation of a ligand. Hence, potassium is protected by an attack from the fellow metal cation sodium in the K@GG^Ⴈ+^ composition, which falls in line with experimental data. On the other hand, the presence of additional nucleic bases adds to the heterogeneity of structure and function of the quadruplexes with arising novel interactions with the constituents. Furthermore, the metal cation rivalry between the native potassium and the less observed sodium in addition to the alien strontium strongly correlates with the balance between electronic and solvation effects. Hence, the replacement of K^+^ with Sr^2+^ occurs spontaneously in the nonpolar environment characterized by ε = 2, whereas this reaction becomes thermodynamically unfavorable with the enhanced polarity of the medium, where the desolvation penalty for the doubly charged strontium increases substantially. A thought-provoking trend is observed in the case of the Na^+^/K^+^ competition in the M@AG^Ⴈ+^ complex: the outer sodium effectively substitutes its contender only when its interaction with the G-layer is increased due to a migration to the plane of the tetrad. Consequently, the arising electronic interaction with the lone pairs of the O-atom from the carbonyl groups compensates for the high desolvation penalty of the small cation. This outcome is attenuated, nonetheless, by enhancing the polarity of the surrounding medium. The computations reveal that the dielectric constant of the environment correlating with exposure to the aqueous environment in the cellular interior has a crucial role, not only on the outcome in metal cation rivalry, but also on the ligand addition to these structures. In combination with previously reported cytotoxic analysis, the presented results provide further strong evidence at an atomic level of the complexation ability of 4-(3,4-dihydroisoquinolin-2-yl)-2-(quinolin-2-yl)quinazoline to the K@GG^Ⴈ+^ and K@AG^Ⴈ+^ compositions, thus providing a solid premise for its application as an anticancer drug. Yet, the presented DFT calculations, although reliable, shed light only on the static aspect of the intricate biochemical processes under study. Hence, as a future perspective, the utilization of MD simulations as an additional approach to the problem and/or the assessment of the effect of trivalent metal cations would undoubtedly add to our understanding of ligand/metal/quadruplex recognition in non-canonical nucleic acids structures.

## Data Availability

The data presented in this study are available on request from the corresponding author.

## Supplementary Materials

The following supporting information can be downloaded at: https://www.mdpi.com/article/10.3390/molecules28166109/s1, Figure S1: Structures and relative stabilities of the hydrated Na^+^, K^+^, and Sr^2+^ ion clusters: Na(H_2_O)_6_^Ⴈ+^ (A), K(H_2_O)_6_^Ⴈ+^ (B), and Sr(H_2_O)_8_^Ⴈ2+^ (C); Structures and relative stabilities of rotamers of Ligand L. All structures are optimized at wb97xd/6-31g(d,p) level of theory; Table S1: ∆H, T∆S, ∆G^1^, ∆G^2^, ∆G^4^, and ∆G^30^ values in kcal mol^−1^ calculated at the wB97XD/6-31G(d,p)//wB97XD/6-31G(d,p) and wB97XD/6-31+G(d,p)//wB97XD/6-31G(d,p) levels of theory for reaction R1: substitution of K^+^ by Na^+^; reaction R2: substitution of K^+^ by Sr^2+^; reaction R3: addition of the ligand 4-(3,4-dihydroisoquinolin-2-yl)-2-(quinolin-2-yl)quinazoline L to the K@GQ^Ⴈ+^ constructs; methodology adopted for implicit solvent calculations; starting geometries for K@GG^Ⴈ+^, K@AG^Ⴈ+^, K@CG^Ⴈ+^, K@TG^Ⴈ+^, and K@UG^Ⴈ+^ optimization. References [39,42,63] are cited in Supplementary Materials.
