# Supplementary material for: Theoretical Assessment of the Ligand/Metal/Quadruplex Recognition in the Non-Canonical Nucleic Acids Structures
Source: Molecules. 2023 Aug 17;28(16):6109. doi: 10.3390/molecules28166109 (PMC10459790; doi:10.3390/molecules28166109)
Supplement: Supplementary file 1 [file molecules-28-06109-s001.zip › molecules-2518020-supplementary.pdf]

Supplementary materials

# Theoretical assessment of the ligand/metal/quadruplex recognition in the non-canonical nucleic acids structures

**Nikoleta Kircheva [1], Stefan Dobrev [1], Vladislava Petkova [1], Snezhana Bakalova [2], Jose Kaneti [2], Silvia Angelova [1,*]**

[1] Institute of Optical Materials and Technologies "Acad. J. Malinowski", Bulgarian Academy of Sciences, 1113 Sofia, Bulgaria; nkircheva@iomt.bas.bg (N. K.), sdobrev@iomt.bas.bg (S. D.), vpetkova@iomt.bas.bg (V. P.)
[2] Institute of Organic Chemistry with Centre of Phytochemistry, Bulgarian Academy of Sciences, 1113 Sofia, Bulgaria; Snezhana.Bakalova@orgchm.bas.bg (S. B.), Jose.Kaneti@orgchm.bas.bg (J. K.)

Correspondence: sea@iomt.bas.bg

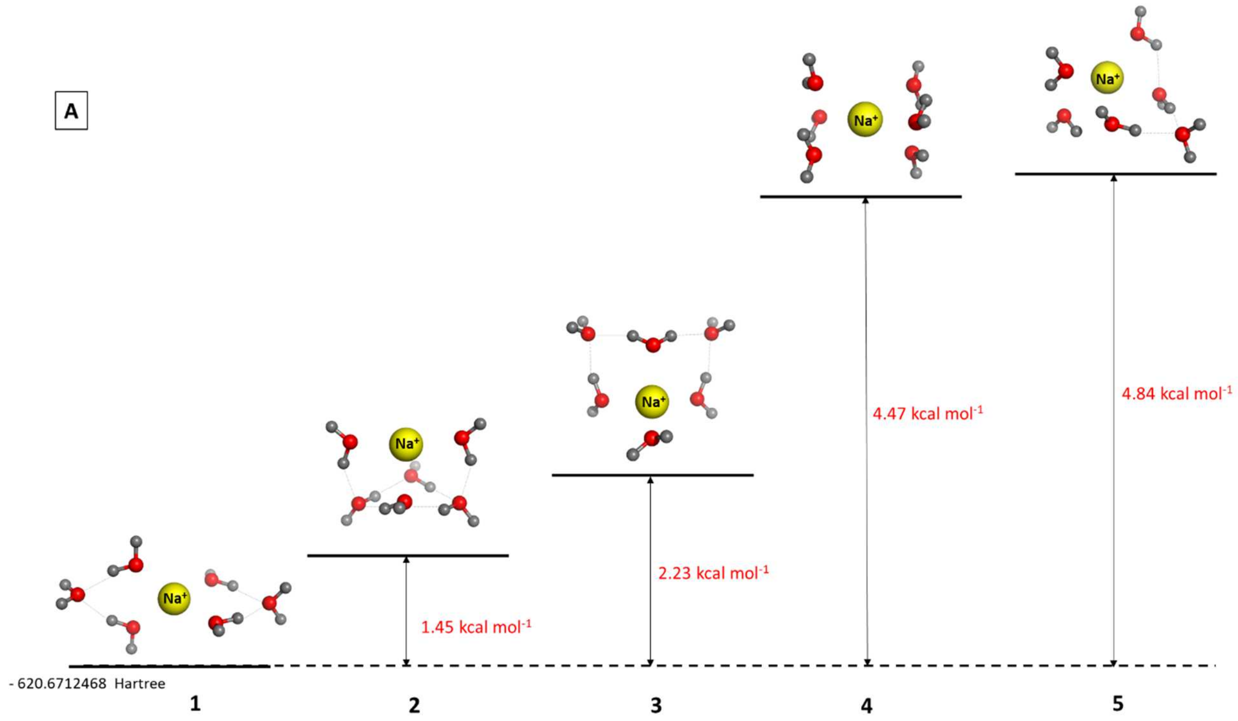

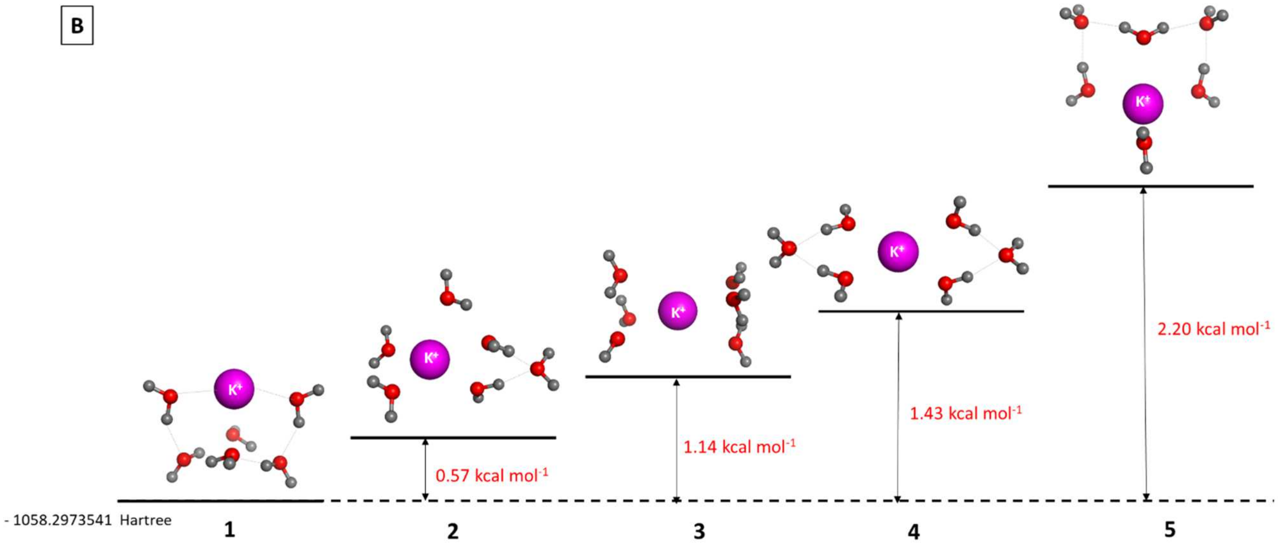

B
K+
0.57 kcal mol-1
1.14 kcal mol-1
1.43 kcal mol-1
2.20 kcal mol-1
- 1058.2973541 Hartree
1
2
3
4
5


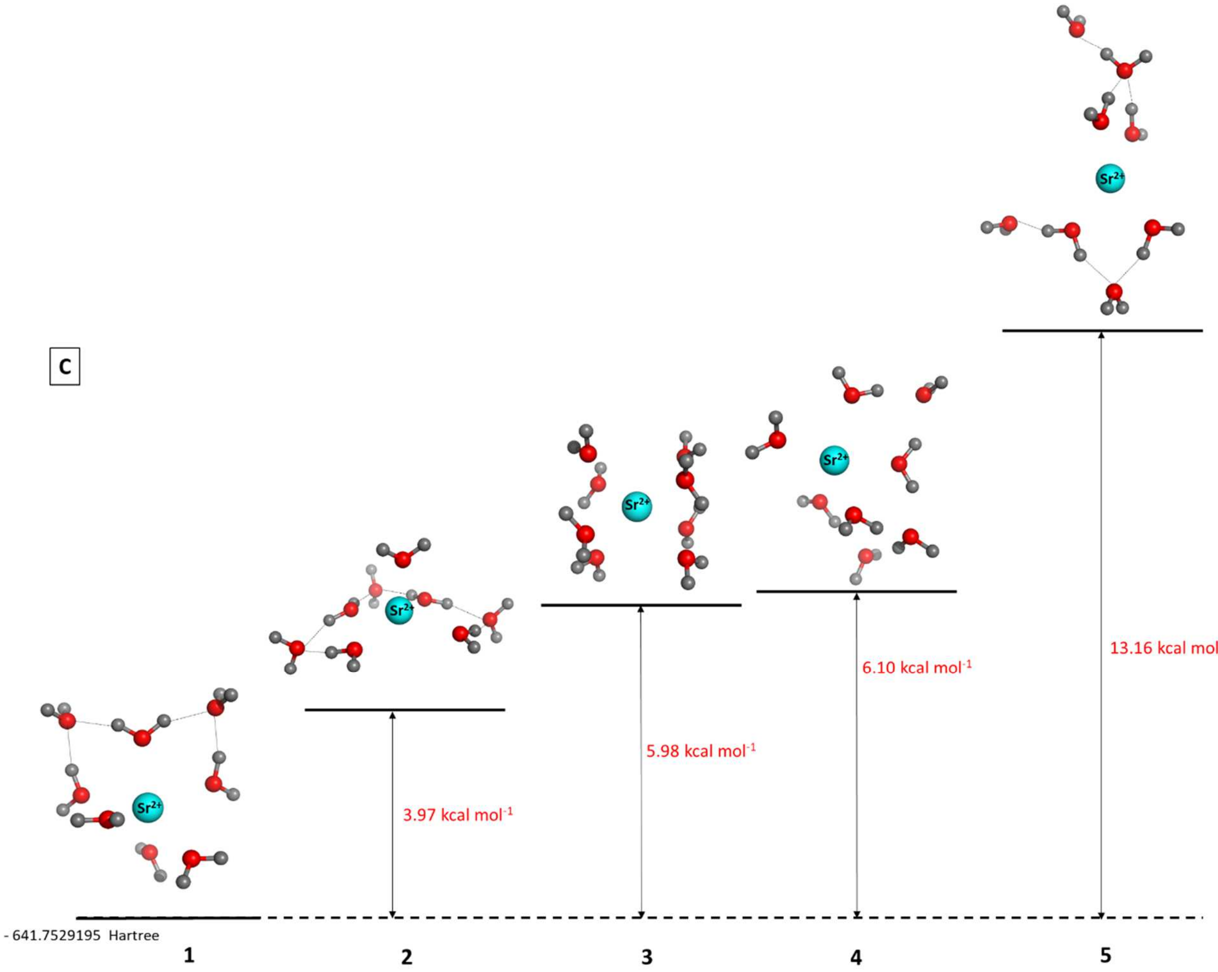

C
Sr2+
3.97 kcal mol-1
5.98 kcal mol-1
6.10 kcal mol-1
13.16 kcal mol-1
- 641.7529195 Hartree
1
2
3
4
5

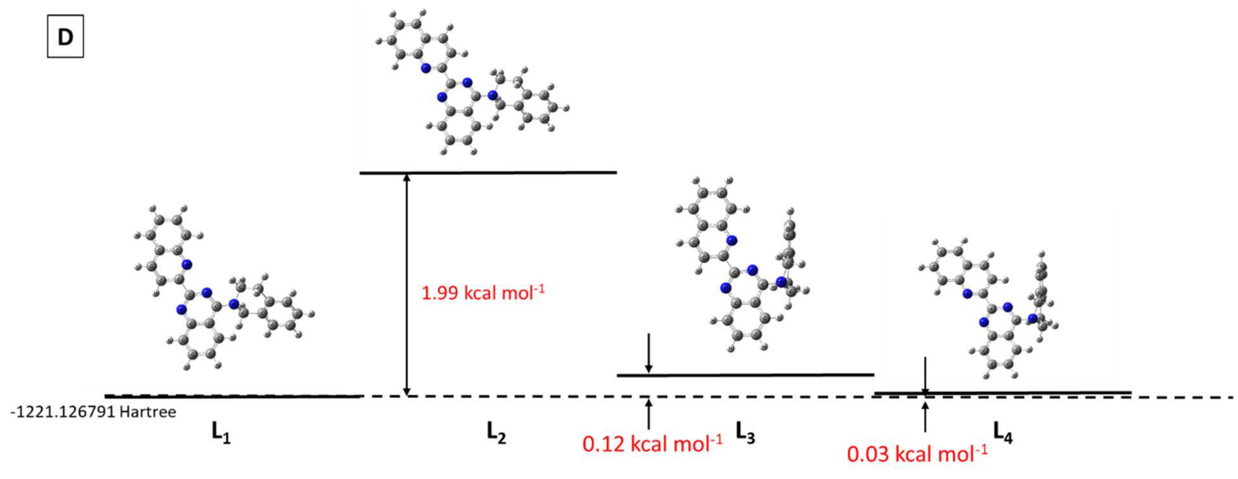


**Figure S1.** Structures and relative stabilities of the hydrated $Na^+$, $K^+$ and $Sr^{2+}$ ion clusters: $Na(H_2O)_6]^{1+}$ (A), $K(H_2O)_6]^{1+}$ (B) and $Sr(H_2O)_8]^{2+}$ (C); Structures and relative stabilities of rotamers of Ligand L. All structures are optimized at wb97xd/6-31g(d,p) level of theory.

**Table S1.** ΔH, TΔS, $\Delta G^1$, $\Delta G^2$, $\Delta G^4$, and $\Delta G^{30}$ values in kcal $mol^{-1}$ calculated at the **wB97XD/6-31G(d,p)//wB97XD/6-31G(d,p)** and **wB97XD/6-31+G(d,p)//wB97XD/6-31G(d,p)** levels of theory for reaction **R1**: substitution of $K^+$ by $Na^+$; reaction **R2**: substitution of $K^+$ by $Sr^{2+}$; reaction **R3**: addition of the ligand 4-(3,4-dihydroisoquinolin-2-yl)-2-(quinolin-2-yl)quinazoline L to the $K@GQ^{1+}$ constructs.

| | | **wB97XD/6-31G(d,p)//wB97XD/6-31G(d,p)** | | | | | | **wB97XD/6-31+G(d,p)//wB97XD/6-31G(d,p)** | | | | | |
|---|---|---|---|---|---|---|---|---|---|---|---|---|---|
| | | **ΔH** | **TΔS** | **$\Delta G^1$** | **$\Delta G^2$** | **$\Delta G^4$** | **$\Delta G^{30}$** | **ΔH** | **TΔS** | **$\Delta G^1$** | **$\Delta G^2$** | **$\Delta G^4$** | **$\Delta G^{30}$** |
| **GG** | **R1** | -4.4 | -7.0 | 2.6 | 3.0 | 3.8 | 4.2 | -3.1 | -7.0 | 3.9 | 5.3 | 5.9 | 6.4 |
| | **R2** | --11.3 | 9.7 | -21.0 | -7.5 | 2.8 | 13.2 | -21.8 | 9.7 | -31.5 | -13.9 | -3.2 | 7.5 |
| | **R3** | -39.4 | -19.2 | -20.2 | -2.4 | -1.2 | -0.8 | -36.8 | -19.2 | -17.6 | -0.1 | 2.1 | 3.4 |
| **AG** | **R1** | -9.2 | -7.5 | -1.7 | -1.9 | -0.7 | 0.4 | -8.7 | -7.5 | -1.3 | -1.1 | -0.4 | 0.6 |
| | **R2** | 13.4 | 11.6 | 1.8 | 1.3 | 11.7 | 20.2 | 2.8 | 11.6 | -8.9 | -0.9 | 6.6 | 13.0 |
| | **R3** | -40.4 | -18.3 | -22.1 | -4.9 | -2.8 | -1.3 | -37.3 | -18.3 | -19.0 | -2.8 | 0.2 | 2.6 |
| **CG** | **R1** | -5.8 | -8.3 | 2.5 | 1.3 | 2.3 | 3.1 | -4.2 | -8.3 | 4.1 | 3.1 | 3.6 | 4.7 |
| | **R2** | -4.4 | 12.5 | -16.9 | -9.5 | -0.3 | 8.5 | -15.4 | 12.5 | -27.9 | -15.5 | -6.7 | 2.4 |
| | **R3** | -31.9 | -17.7 | -14.2 | -0.7 | 1.6 | 0.7 | -30.2 | -17.7 | -12.5 | 1.4 | 4.0 | 4.1 |
| **TG** | **R1** | -2.8 | -6.1 | 3.3 | 3.5 | 4.4 | 4.9 | -1.6 | -6.1 | 4.5 | 5.7 | 6.2 | 6.8 |
| | **R2** | -5.3 | 9.6 | -14.9 | -6.5 | 2.0 | 12.1 | -15.9 | 9.6 | -25.5 | -10.6 | -2.2 | 6.4 |
| | **R3** | -35.7 | -19.2 | -16.5 | -2.4 | -1.3 | -1.3 | -32.3 | -19.2 | -13.1 | 1.6 | 3.5 | 4.1 |
| **UG** | R1 | -2.3 | -7.6 | 5.3 | 5.2 | 6.1 | 6.8 | -0.6 | -7.6 | 7.0 | 7.7 | 8.2 | 8.9 |
| | R2 | -5.8 | 8.0 | -13.8 | -3.5 | 5.6 | 15.3 | -17.3 | 8.0 | -25.3 | -10.5 | -1.2 | 8.9 |
| | R3 | -37.7 | -21.6 | -16.1 | -3.0 | -1.1 | 0.3 | -34.4 | -21.6 | -12.8 | 0.8 | 3.6 | 5.8 |

**Methodology adopted for implicit solvent calculations**

For the implicit solvation calculations, the "generic" solvent option was used with Eps (2/4/30) and EpsInf (2) values specified. As we are aware that 7 parameters are needed to define a solvent for SMD, additional calculations were performed with the following pre-defined solvents (n-dodecane, $\varepsilon$=2.0060; diethylether, $\varepsilon$= 4.2400; propanonitrile: $\varepsilon$=29.324) for reaction (1). These particular solvents were chosen as they are widely accepted and applied in other articles when simulating biological environment [39,42]. The results show that for each couple (incomplete specification of generic solvent/pre-defined solvent with close $\varepsilon$ value) there is a difference of about 0.5 kcal mol$^{-1}$ for the wB97XD/6-31G(d,p)//wB97XD/6-31G(d,p) calculations: $\Delta G^{2}$= 0.3 vs 0.1/ $\Delta G^{4}$= 0.6 vs 1.1/ $\Delta G^{30}$= 1.0 vs 1.5, respectively, and about 0.1-0.2 kcal mol$^{-1}$ for the wB97XD/6-311G(d,p)//wB97XD/6-31G(d,p) calculations: $\Delta G^{2}$= 0.9 vs 1.0/ $\Delta G^{4}$= 1.3 vs 1.5/ $\Delta G^{30}$= 1.7 vs 1.9 kcal mol$^{-1}$, respectively. Note that these differences do not change the observed trends, but rather bring stronger evidence to the drawn conclusions. As we have further performed higher–level calculations, we believe that the definition of the solvent does not play a significant role in the obtained results, as seen in our previous work where the same scheme was applied [63].

**Starting geometries for K@GG$^{1+}$, K@AG$^{1+}$, K@CG$^{1+}$, K@TG$^{1+}$ and K@UG$^{1+}$ optimization**

**K@GG$^{1+}$:**

| | | | |
|---|---|---|---|
| 7 | -6.998881000 | -0.528519000 | -1.146479000 |
| 6 | -6.706627000 | 0.764359000 | -1.343028000 |
| 6 | -5.487695000 | 1.334703000 | -1.673569000 |
| 6 | -4.353361000 | 0.487503000 | -1.800931000 |
| 7 | -4.681373000 | -0.850984000 | -1.592501000 |
| 6 | -5.941179000 | -1.313536000 | -1.293625000 |
| 7 | -7.571843000 | 1.822026000 | -1.254280000 |
| 6 | -6.851961000 | 2.958665000 | -1.536506000 |
| 7 | -5.599285000 | 2.705124000 | -1.792795000 |
| 7 | -6.087061000 | -2.635588000 | -1.136603000 |
| 7 | -1.746917290 | 3.138109430 | -2.781711460 |
| 6 | -0.406634290 | 2.790648430 | -2.940509460 |
| 6 | 0.394679710 | 3.917967430 | -3.270385460 |
| 6 | -0.217987290 | 5.156081430 | -3.381864460 |
| 7 | -1.514944290 | 5.463217430 | -3.241371460 |
| 6 | -2.253389290 | 4.406757430 | -2.934457460 |
| 7 | 1.754565710 | 4.017899430 | -3.481768460 |
| 6 | 1.961789710 | 5.284360430 | -3.710403460 |
| 7 | 0.803622710 | 6.024509430 | -3.660937460 |
| 8 | -0.036242290 | 1.621145430 | -2.789458460 |
| 7 | -3.573188290 | 4.562983430 | -2.765036460 |
| 7 | 2.297365710 | 0.078986430 | -3.243503460 |
| 6 | 3.557549710 | 0.532026430 | -3.554466460 |
| 7 | 4.621855710 | -0.249714570 | -3.633755460 |
| 6 | 4.338291710 | -1.529662570 | -3.353798460 |
| 6 | 3.108489710 | -2.097320570 | -3.059965460 |
| 6 | 1.969993710 | -1.251077570 | -2.987489460 |
| 7 | 3.234011710 | -3.451204570 | -2.820132460 |
| 6 | 4.508236710 | -3.692733570 | -2.959613460 |
| 7 | 5.223656710 | -2.570723570 | -3.294942460 |
| 7 | 3.691567710 | 1.844865430 | -3.795403460 |
| 8 | 0.803899710 | -1.569211570 | -2.722222460 |
| 7 | -0.718155290 | -3.913770570 | -2.361722460 |
| 6 | -2.022466290 | -3.558617570 | -2.034129460 |
| 6 | -2.842777290 | -4.699804570 | -1.821026460 |
| 6 | -2.267838290 | -5.956546570 | -1.928850460 |
| 7 | -1.003849290 | -6.274090570 | -2.233840460 |

| | | | |
|---|---|---|---|
| 6 | -0.249566290 | -5.203271570 | -2.440040460 |
| 7 | -4.172354290 | -4.794126570 | -1.462649460 |
| 6 | -4.397375290 | -6.074112570 | -1.356074460 |
| 7 | -3.280489290 | -6.828370570 | -1.626056460 |
| 8 | -2.352713290 | -2.368476570 | -1.950174460 |
| 7 | 1.040621710 | -5.367921570 | -2.761836460 |
| 1 | -0.069379290 | -3.124832570 | -2.516593460 |
| 1 | 1.739252710 | -4.615777570 | -2.748306460 |
| 1 | 1.364772710 | -6.319818570 | -2.743201460 |
| 1 | 1.515600710 | 0.746225430 | -3.163545460 |
| 1 | 4.632011710 | 2.159533430 | -3.958899460 |
| 1 | 2.953149710 | 2.538934430 | -3.644920460 |
| 1 | 0.702983710 | 7.010037430 | -3.841967460 |
| 1 | -2.379980290 | 2.360671430 | -2.536709460 |
| 1 | -3.914215290 | 5.502957430 | -2.866726460 |
| 1 | -4.208905290 | 3.835885430 | -2.419380460 |
| 1 | -3.901561000 | -1.520793000 | -1.705539000 |
| 1 | -7.026326000 | -2.950500000 | -0.966782000 |
| 1 | -5.341260000 | -3.329819000 | -1.265824000 |
| 1 | -7.306822000 | 3.937801000 | -1.530852000 |
| 1 | -5.340106290 | -6.526186570 | -1.086069460 |
| 1 | 4.982258710 | -4.653590570 | -2.827294460 |
| 1 | 2.920292710 | 5.737308430 | -3.915503460 |
| 1 | -8.558281000 | 1.758595000 | -1.062052000 |
| 1 | -3.213347290 | -7.833052570 | -1.643899460 |
| 1 | 6.219962710 | -2.500603570 | -3.420375460 |
| 19 | -0.886387290 | -0.250248570 | -0.980395460 |
| 7 | -4.335056290 | -4.433838570 | 1.878950540 |
| 6 | -5.032706290 | -3.293713570 | 1.960617540 |
| 6 | -4.597541290 | -1.994997570 | 1.745592540 |
| 6 | -3.250291290 | -1.789110570 | 1.342227540 |
| 7 | -2.552394290 | -2.990571570 | 1.237908540 |
| 6 | -3.078764290 | -4.231687570 | 1.509967540 |
| 7 | -6.358687290 | -3.169398570 | 2.276388540 |
| 6 | -6.660337290 | -1.829563570 | 2.235012540 |
| 7 | -5.629009290 | -1.095640570 | 1.923547540 |
| 8 | -2.690483290 | -0.712854570 | 1.093786540 |
| 7 | -2.254114290 | -5.282055570 | 1.411457540 |
| 7 | -3.432058290 | 2.022927430 | 1.013563540 |
| 6 | -2.313057290 | 2.712864430 | 0.562214540 |
| 6 | -2.573018290 | 4.100946430 | 0.408431540 |
| 6 | -3.838693290 | 4.575039430 | 0.710231540 |
| 7 | -4.900882290 | 3.893451430 | 1.155489540 |
| 6 | -4.646839290 | 2.599832430 | 1.296444540 |
| 7 | -1.735932290 | 5.129167430 | 0.030026540 |
| 6 | -2.478968290 | 6.199686430 | 0.086545540 |
| 7 | -3.764169290 | 5.925057430 | 0.491056540 |
| 8 | -1.249658290 | 2.119366430 | 0.344462540 |
| 7 | -5.618667290 | 1.791719430 | 1.736081540 |
| 7 | 1.452563710 | 2.733455430 | -0.139139460 |
| 6 | 2.001535710 | 3.984645430 | -0.293345460 |
| 7 | 3.286150710 | 4.204480430 | -0.517352460 |
| 6 | 3.992837710 | 3.065919430 | -0.595636460 |
| 6 | 3.547066710 | 1.764619430 | -0.436762460 |
| 6 | 2.167506710 | 1.537719430 | -0.193016460 |

| | | | |
|---|---|---|---|
| 7 | 4.573948710 | 0.865815430 | -0.613816460 |
| 6 | 5.618020710 | 1.598496430 | -0.876858460 |
| 7 | 5.325274710 | 2.942898430 | -0.881936460 |
| 7 | 1.170838710 | 5.035398430 | -0.185324460 |
| 8 | 1.592097710 | 0.451909430 | -0.049192460 |
| 7 | 2.278826710 | -2.308266570 | 0.112908540 |
| 6 | 1.113404710 | -3.048422570 | 0.252867540 |
| 6 | 1.383064710 | -4.424806570 | 0.470335540 |
| 6 | 2.703813710 | -4.826594570 | 0.585786540 |
| 7 | 3.813793710 | -4.089267570 | 0.482633540 |
| 6 | 3.554821710 | -2.814987570 | 0.224144540 |
| 7 | 0.523766710 | -5.481985570 | 0.683869540 |
| 6 | 1.303483710 | -6.504884570 | 0.903369540 |
| 7 | 2.634826710 | -6.168660570 | 0.856053540 |
| 8 | 0.003190710 | -2.500778570 | 0.203829540 |
| 7 | 4.571721710 | -1.963326570 | 0.030590540 |
| 1 | 2.149646710 | -1.289415570 | 0.025389540 |
| 1 | 4.413955710 | -0.967419570 | -0.143394460 |
| 1 | 5.408373710 | -2.160413570 | 0.561515540 |
| 1 | 0.437711710 | 2.632376430 | 0.017187540 |
| 1 | 1.587004710 | 5.929785430 | -0.379788460 |
| 1 | 0.148531710 | 4.962957430 | -0.164340460 |
| 1 | -4.509911290 | 6.585773430 | 0.637204540 |
| 1 | -3.295374290 | 1.008945430 | 1.145089540 |
| 1 | -6.505830290 | 2.231901430 | 1.906427540 |
| 1 | -5.541513290 | 0.769388430 | 1.790249540 |
| 1 | -1.576458290 | -2.913136570 | 0.901487540 |
| 1 | -2.681156290 | -6.179917570 | 1.561266540 |
| 1 | -1.286227290 | -5.245626570 | 1.067499540 |
| 1 | -7.654148290 | -1.461110570 | 2.441248540 |
| 1 | 0.975332710 | -7.514859570 | 1.100016540 |
| 1 | 6.614375710 | 1.227513430 | -1.065243460 |
| 1 | -2.158699290 | 7.200468430 | -0.161777460 |
| 1 | -6.977677290 | -3.926304570 | 2.517248540 |
| 1 | 3.423141710 | -6.775026570 | 1.014876540 |
| 1 | 5.973124710 | 3.702407430 | -1.010903460 |
| 8 | -3.188013290 | 0.819136430 | -2.054956460 |

**K@AG$^{T+}$:**

| | | | |
|---|---|---|---|
| 7 | 3.822000000 | 6.806000000 | -4.639000000 |
| 6 | 4.073000000 | 6.527000000 | -3.352000000 |
| 7 | 3.002000000 | 6.347000000 | -2.624000000 |
| 6 | 1.965000000 | 6.465000000 | -3.565000000 |
| 6 | 0.555000000 | 6.350000000 | -3.547000000 |
| 7 | -0.157000000 | 5.979000000 | -2.505000000 |
| 7 | -0.167000000 | 6.511000000 | -4.663000000 |
| 6 | 0.472000000 | 6.761000000 | -5.798000000 |
| 7 | 1.781000000 | 6.877000000 | -5.975000000 |
| 6 | 2.471000000 | 6.723000000 | -4.810000000 |
| 1 | 5.125000000 | 6.488000000 | -3.123000000 |
| 1 | -1.140000000 | 5.867000000 | -2.753000000 |
| 1 | 0.307000000 | 5.415000000 | -1.804000000 |
| 1 | -0.114000000 | 6.899000000 | -6.694000000 |
| 7 | 6.074000000 | 3.476000000 | -2.459000000 |
| 6 | 5.289000000 | 3.372000000 | -3.577000000 |

| | | | |
|---|---|---|---|
| 7 | 4.004000000 | 3.394000000 | -3.345000000 |
| 6 | 3.935000000 | 3.505000000 | -1.951000000 |
| 6 | 2.824000000 | 3.672000000 | -1.057000000 |
| 8 | 1.642000000 | 3.858000000 | -1.333000000 |
| 7 | 3.173000000 | 3.649000000 | 0.278000000 |
| 6 | 4.453000000 | 3.644000000 | 0.714000000 |
| 7 | 4.641000000 | 3.652000000 | 2.006000000 |
| 7 | 5.519000000 | 3.608000000 | -0.081000000 |
| 6 | 5.198000000 | 3.529000000 | -1.405000000 |
| 1 | 5.711000000 | 3.331000000 | -4.570000000 |
| 1 | 2.408000000 | 3.677000000 | 0.931000000 |
| 1 | 3.871000000 | 3.570000000 | 2.671000000 |
| 1 | 5.601000000 | 3.572000000 | 2.282000000 |
| 7 | -4.797000000 | 6.235000000 | -4.171000000 |
| 6 | -3.525000000 | 5.846000000 | -4.341000000 |
| 7 | -2.831000000 | 5.773000000 | -3.234000000 |
| 6 | -3.781000000 | 6.118000000 | -2.255000000 |
| 6 | -3.791000000 | 6.254000000 | -0.848000000 |
| 7 | -2.757000000 | 6.050000000 | -0.064000000 |
| 7 | -4.917000000 | 6.549000000 | -0.186000000 |
| 6 | -6.032000000 | 6.716000000 | -0.883000000 |
| 7 | -6.176000000 | 6.636000000 | -2.199000000 |
| 6 | -5.003000000 | 6.341000000 | -2.827000000 |
| 1 | -3.283000000 | 5.644000000 | -5.373000000 |
| 1 | -3.024000000 | 6.040000000 | 0.920000000 |
| 1 | -1.950000000 | 5.572000000 | -0.429000000 |
| 1 | -6.934000000 | 6.967000000 | -0.347000000 |
| 7 | -2.498000000 | 2.852000000 | -6.524000000 |
| 6 | -3.599000000 | 2.712000000 | -5.719000000 |
| 7 | -3.347000000 | 2.762000000 | -4.439000000 |
| 6 | -1.958000000 | 2.934000000 | -4.397000000 |
| 6 | -1.048000000 | 3.104000000 | -3.303000000 |
| 8 | -1.302000000 | 3.190000000 | -2.109000000 |
| 7 | 0.276000000 | 3.206000000 | -3.677000000 |
| 6 | 0.680000000 | 3.269000000 | -4.966000000 |
| 7 | 1.959000000 | 3.413000000 | -5.173000000 |
| 7 | -0.130000000 | 3.172000000 | -6.015000000 |
| 6 | -1.437000000 | 2.997000000 | -5.669000000 |
| 1 | -4.597000000 | 2.613000000 | -6.123000000 |
| 1 | 0.932000000 | 3.303000000 | -2.920000000 |
| 1 | 2.631000000 | 3.399000000 | -4.406000000 |
| 1 | 2.233000000 | 3.377000000 | -6.138000000 |
| 7 | -4.516000000 | 6.220000000 | 4.533000000 |
| 6 | -4.655000000 | 5.834000000 | 3.257000000 |
| 7 | -3.545000000 | 5.857000000 | 2.565000000 |
| 6 | -2.596000000 | 6.260000000 | 3.519000000 |
| 6 | -1.201000000 | 6.492000000 | 3.533000000 |
| 7 | -0.408000000 | 6.356000000 | 2.493000000 |
| 7 | -0.560000000 | 6.812000000 | 4.664000000 |
| 6 | -1.268000000 | 6.911000000 | 5.781000000 |
| 7 | -2.576000000 | 6.740000000 | 5.923000000 |
| 6 | -3.182000000 | 6.422000000 | 4.744000000 |
| 1 | -5.666000000 | 5.555000000 | 3.005000000 |
| 1 | 0.578000000 | 6.427000000 | 2.744000000 |
| 1 | -0.744000000 | 5.887000000 | 1.669000000 |

| | | | |
|---|---|---|---|
| 1 | -0.753000000 | 7.183000000 | 6.689000000 |
| 7 | -6.498000000 | 2.615000000 | 2.140000000 |
| 6 | -5.700000000 | 2.592000000 | 3.254000000 |
| 7 | -4.428000000 | 2.766000000 | 3.017000000 |
| 6 | -4.381000000 | 2.888000000 | 1.623000000 |
| 6 | -3.291000000 | 3.119000000 | 0.721000000 |
| 8 | -2.117000000 | 3.339000000 | 0.985000000 |
| 7 | -3.647000000 | 3.112000000 | -0.611000000 |
| 6 | -4.925000000 | 2.999000000 | -1.040000000 |
| 7 | -5.112000000 | 2.984000000 | -2.332000000 |
| 7 | -5.976000000 | 2.849000000 | -0.240000000 |
| 6 | -5.642000000 | 2.790000000 | 1.082000000 |
| 1 | -6.106000000 | 2.481000000 | 4.249000000 |
| 1 | -2.887000000 | 3.177000000 | -1.266000000 |
| 1 | -4.334000000 | 2.956000000 | -2.991000000 |
| 1 | -6.058000000 | 2.798000000 | -2.609000000 |
| 7 | 4.216000000 | 6.835000000 | 4.143000000 |
| 6 | 2.933000000 | 6.483000000 | 4.302000000 |
| 7 | 2.254000000 | 6.408000000 | 3.186000000 |
| 6 | 3.230000000 | 6.690000000 | 2.214000000 |
| 6 | 3.268000000 | 6.783000000 | 0.803000000 |
| 7 | 2.235000000 | 6.606000000 | 0.010000000 |
| 7 | 4.412000000 | 7.035000000 | 0.153000000 |
| 6 | 5.519000000 | 7.192000000 | 0.865000000 |
| 7 | 5.639000000 | 7.145000000 | 2.185000000 |
| 6 | 4.449000000 | 6.895000000 | 2.800000000 |
| 1 | 2.666000000 | 6.324000000 | 5.334000000 |
| 1 | 2.485000000 | 6.584000000 | -0.979000000 |
| 1 | 1.364000000 | 6.268000000 | 0.376000000 |
| 1 | 6.435000000 | 7.412000000 | 0.338000000 |
| 7 | 2.078000000 | 3.265000000 | 6.233000000 |
| 6 | 3.190000000 | 3.286000000 | 5.432000000 |
| 7 | 2.938000000 | 3.371000000 | 4.154000000 |
| 6 | 1.538000000 | 3.390000000 | 4.108000000 |
| 6 | 0.619000000 | 3.503000000 | 3.015000000 |
| 8 | 0.862000000 | 3.702000000 | 1.832000000 |
| 7 | -0.707000000 | 3.393000000 | 3.378000000 |
| 6 | -1.125000000 | 3.309000000 | 4.661000000 |
| 7 | -2.411000000 | 3.206000000 | 4.851000000 |
| 7 | -0.313000000 | 3.279000000 | 5.714000000 |
| 6 | 1.009000000 | 3.312000000 | 5.376000000 |
| 1 | 4.190000000 | 3.275000000 | 5.840000000 |
| 1 | -1.367000000 | 3.384000000 | 2.618000000 |
| 1 | -3.059000000 | 3.094000000 | 4.071000000 |
| 1 | -2.677000000 | 3.035000000 | 5.804000000 |
| 1 | -5.249246750 | 6.337625580 | 5.202711450 |
| 1 | -5.466619800 | 6.413307880 | -4.891982400 |
| 1 | 4.497338720 | 7.032189290 | -5.340965820 |
| 1 | 4.877126050 | 7.018801990 | 4.870412650 |
| 1 | -2.474266750 | 2.848941910 | -7.523713650 |
| 1 | -7.493218640 | 2.523608720 | 2.105540090 |
| 1 | 2.051950000 | 3.224036670 | 7.231821010 |
| 1 | 7.072758960 | 3.506719160 | -2.419796950 |
| 19 | -0.489779900 | 4.747027400 | -0.112638490 |

**K@CG$^{1+}$:**

| | | | |
|---|---|---|---|
| 7 | 0.061000000 | -0.206000000 | 5.816000000 |
| 6 | -0.026000000 | -0.067000000 | 4.421000000 |
| 8 | -1.110000000 | -0.066000000 | 3.845000000 |
| 7 | 1.131000000 | 0.075000000 | 3.704000000 |
| 6 | 2.329000000 | 0.074000000 | 4.310000000 |
| 7 | 3.420000000 | 0.218000000 | 3.562000000 |
| 6 | 2.449000000 | -0.089000000 | 5.734000000 |
| 6 | 1.293000000 | -0.224000000 | 6.432000000 |
| 1 | 3.318000000 | 0.321000000 | 2.562000000 |
| 1 | 4.332000000 | 0.215000000 | 3.992000000 |
| 1 | 3.398000000 | -0.109000000 | 6.250000000 |
| 1 | 1.305000000 | -0.356000000 | 7.503000000 |
| 7 | -4.648000000 | -3.360000000 | 4.393000000 |
| 6 | -3.440000000 | -3.190000000 | 4.984000000 |
| 7 | -2.453000000 | -2.978000000 | 4.163000000 |
| 6 | -3.067000000 | -3.029000000 | 2.914000000 |
| 6 | -2.512000000 | -2.896000000 | 1.601000000 |
| 8 | -1.342000000 | -2.686000000 | 1.303000000 |
| 7 | -3.462000000 | -3.041000000 | 0.586000000 |
| 6 | -4.803000000 | -3.288000000 | 0.823000000 |
| 7 | -5.594000000 | -3.407000000 | -0.232000000 |
| 7 | -5.334000000 | -3.415000000 | 2.050000000 |
| 6 | -4.419000000 | -3.275000000 | 3.049000000 |
| 1 | -3.423000000 | -3.268000000 | 6.061000000 |
| 1 | -3.129000000 | -2.944000000 | -0.364000000 |
| 1 | -5.213000000 | -3.314000000 | -1.164000000 |
| 1 | -6.572000000 | -3.613000000 | -0.092000000 |
| 7 | -0.146000000 | 0.160000000 | -6.184000000 |
| 6 | -0.034000000 | 0.213000000 | -4.786000000 |
| 8 | 1.060000000 | 0.264000000 | -4.233000000 |
| 7 | -1.180000000 | 0.219000000 | -4.038000000 |
| 6 | -2.388000000 | 0.164000000 | -4.620000000 |
| 7 | -3.467000000 | 0.183000000 | -3.842000000 |
| 6 | -2.531000000 | 0.080000000 | -6.049000000 |
| 6 | -1.385000000 | 0.083000000 | -6.777000000 |
| 1 | -3.346000000 | 0.227000000 | -2.839000000 |
| 1 | -4.386000000 | 0.139000000 | -4.253000000 |
| 1 | -3.488000000 | 0.009000000 | -6.545000000 |
| 1 | -1.404000000 | 0.021000000 | -7.854000000 |
| 7 | 4.772000000 | -2.797000000 | -5.027000000 |
| 6 | 3.558000000 | -2.599000000 | -5.596000000 |
| 7 | 2.574000000 | -2.461000000 | -4.757000000 |
| 6 | 3.197000000 | -2.585000000 | -3.518000000 |
| 6 | 2.641000000 | -2.544000000 | -2.199000000 |
| 8 | 1.463000000 | -2.384000000 | -1.895000000 |
| 7 | 3.597000000 | -2.718000000 | -1.193000000 |
| 6 | 4.946000000 | -2.916000000 | -1.449000000 |
| 7 | 5.751000000 | -3.062000000 | -0.405000000 |
| 7 | 5.474000000 | -2.967000000 | -2.685000000 |
| 6 | 4.553000000 | -2.794000000 | -3.674000000 |
| 1 | 3.529000000 | -2.600000000 | -6.675000000 |
| 1 | 3.258000000 | -2.682000000 | -0.240000000 |
| 1 | 5.376000000 | -3.031000000 | 0.533000000 |
| 1 | 6.736000000 | -3.220000000 | -0.561000000 |

| | | | |
|---|---|---|---|
| 7 | -6.031000000 | -0.165000000 | 0.028000000 |
| 6 | -4.632000000 | -0.043000000 | 0.049000000 |
| 8 | -3.981000000 | 0.022000000 | -0.990000000 |
| 7 | -3.998000000 | 0.009000000 | 1.261000000 |
| 6 | -4.688000000 | -0.065000000 | 2.409000000 |
| 7 | -4.021000000 | 0.003000000 | 3.558000000 |
| 6 | -6.118000000 | -0.219000000 | 2.417000000 |
| 6 | -6.733000000 | -0.261000000 | 1.208000000 |
| 1 | -3.016000000 | 0.102000000 | 3.530000000 |
| 1 | -4.516000000 | -0.053000000 | 4.435000000 |
| 1 | -6.702000000 | -0.301000000 | 3.323000000 |
| 1 | -7.802000000 | -0.375000000 | 1.135000000 |
| 7 | -4.608000000 | -3.132000000 | -4.945000000 |
| 6 | -5.205000000 | -3.022000000 | -3.731000000 |
| 7 | -4.389000000 | -2.881000000 | -2.728000000 |
| 6 | -3.137000000 | -2.907000000 | -3.333000000 |
| 6 | -1.830000000 | -2.809000000 | -2.756000000 |
| 8 | -1.544000000 | -2.684000000 | -1.570000000 |
| 7 | -0.805000000 | -2.872000000 | -3.701000000 |
| 6 | -1.028000000 | -3.025000000 | -5.058000000 |
| 7 | 0.040000000 | -3.059000000 | -5.840000000 |
| 7 | -2.249000000 | -3.133000000 | -5.611000000 |
| 6 | -3.261000000 | -3.063000000 | -4.701000000 |
| 1 | -6.284000000 | -3.081000000 | -3.714000000 |
| 1 | 0.140000000 | -2.782000000 | -3.352000000 |
| 1 | 0.965000000 | -2.969000000 | -5.440000000 |
| 1 | -0.080000000 | -3.190000000 | -6.834000000 |
| 7 | 6.031000000 | 0.346000000 | -0.338000000 |
| 6 | 4.628000000 | 0.366000000 | -0.323000000 |
| 8 | 4.001000000 | 0.363000000 | 0.733000000 |
| 7 | 3.962000000 | 0.399000000 | -1.518000000 |
| 6 | 4.631000000 | 0.393000000 | -2.682000000 |
| 7 | 3.934000000 | 0.438000000 | -3.815000000 |
| 6 | 6.068000000 | 0.322000000 | -2.724000000 |
| 6 | 6.712000000 | 0.301000000 | -1.530000000 |
| 1 | 2.925000000 | 0.466000000 | -3.765000000 |
| 1 | 4.410000000 | 0.424000000 | -4.702000000 |
| 1 | 6.632000000 | 0.284000000 | -3.645000000 |
| 1 | 7.786000000 | 0.239000000 | -1.464000000 |
| 7 | 4.762000000 | -3.165000000 | 4.295000000 |
| 6 | 5.344000000 | -2.946000000 | 3.090000000 |
| 7 | 4.515000000 | -2.749000000 | 2.108000000 |
| 6 | 3.272000000 | -2.863000000 | 2.721000000 |
| 6 | 1.957000000 | -2.777000000 | 2.161000000 |
| 8 | 1.663000000 | -2.562000000 | 0.990000000 |
| 7 | 0.945000000 | -2.979000000 | 3.105000000 |
| 6 | 1.187000000 | -3.238000000 | 4.443000000 |
| 7 | 0.131000000 | -3.409000000 | 5.226000000 |
| 7 | 2.417000000 | -3.324000000 | 4.977000000 |
| 6 | 3.414000000 | -3.129000000 | 4.069000000 |
| 1 | 6.424000000 | -2.980000000 | 3.067000000 |
| 1 | -0.007000000 | -2.917000000 | 2.775000000 |
| 1 | -0.802000000 | -3.344000000 | 4.840000000 |
| 1 | 0.272000000 | -3.622000000 | 6.201000000 |
| 19 | 0.118156470 | -1.364916940 | -0.310868660 |

| | | | |
|---|---|---|---|
| 1 | -0.772083400 | -0.291962710 | 6.362426990 |
| 1 | -5.525338940 | -3.515478140 | 4.846985600 |
| 1 | 5.223354140 | -3.320668300 | 5.168452770 |
| 1 | 6.537821510 | 0.364610700 | 0.523850100 |
| 1 | 5.646173490 | -2.919805680 | -5.496829200 |
| 1 | 0.677547360 | 0.178267080 | -6.750953310 |
| 1 | -5.056983590 | -3.240655060 | -5.831909140 |
| 1 | -6.517701500 | -0.182139610 | -0.845400190 |

**K@TG$^{1+}$:**

| | | | |
|---|---|---|---|
| 7 | -4.445000000 | 8.389000000 | -3.596000000 |
| 6 | -3.071000000 | 8.419000000 | -3.364000000 |
| 8 | -2.255000000 | 8.813000000 | -4.182000000 |
| 7 | -2.632000000 | 7.996000000 | -2.135000000 |
| 6 | -3.438000000 | 7.621000000 | -1.084000000 |
| 8 | -2.909000000 | 7.359000000 | -0.009000000 |
| 6 | -4.874000000 | 7.598000000 | -1.387000000 |
| 6 | -5.908000000 | 7.191000000 | -0.342000000 |
| 6 | -5.316000000 | 7.975000000 | -2.616000000 |
| 1 | -1.632000000 | 7.959000000 | -1.993000000 |
| 1 | -5.427000000 | 6.698000000 | 0.502000000 |
| 1 | -6.422000000 | 8.076000000 | 0.034000000 |
| 1 | -6.644000000 | 6.507000000 | -0.768000000 |
| 1 | -6.368000000 | 7.943000000 | -2.863000000 |
| 7 | -2.083000000 | 4.332000000 | -6.097000000 |
| 6 | -3.213000000 | 4.058000000 | -5.372000000 |
| 7 | -3.029000000 | 4.006000000 | -4.080000000 |
| 6 | -1.652000000 | 4.203000000 | -3.948000000 |
| 6 | -0.800000000 | 4.252000000 | -2.797000000 |
| 8 | -1.116000000 | 4.171000000 | -1.621000000 |
| 7 | 0.537000000 | 4.422000000 | -3.086000000 |
| 6 | 1.016000000 | 4.539000000 | -4.344000000 |
| 7 | 2.311000000 | 4.618000000 | -4.475000000 |
| 7 | 0.265000000 | 4.515000000 | -5.440000000 |
| 6 | -1.063000000 | 4.351000000 | -5.181000000 |
| 1 | -4.179000000 | 3.917000000 | -5.826000000 |
| 1 | 1.141000000 | 4.489000000 | -2.285000000 |
| 1 | 2.937000000 | 4.564000000 | -3.674000000 |
| 1 | 2.636000000 | 4.604000000 | -5.425000000 |
| 7 | -4.442000000 | 8.318000000 | 4.516000000 |
| 6 | -4.169000000 | 8.222000000 | 3.151000000 |
| 8 | -4.989000000 | 8.449000000 | 2.276000000 |
| 7 | -2.894000000 | 7.867000000 | 2.789000000 |
| 6 | -1.847000000 | 7.657000000 | 3.655000000 |
| 8 | -0.732000000 | 7.449000000 | 3.187000000 |
| 6 | -2.202000000 | 7.724000000 | 5.077000000 |
| 6 | -1.178000000 | 7.445000000 | 6.170000000 |
| 6 | -3.469000000 | 8.049000000 | 5.449000000 |
| 1 | -2.719000000 | 7.755000000 | 1.799000000 |
| 1 | -0.861000000 | 8.379000000 | 6.634000000 |
| 1 | -1.593000000 | 6.782000000 | 6.932000000 |
| 1 | -0.297000000 | 6.963000000 | 5.749000000 |
| 1 | -3.744000000 | 8.093000000 | 6.494000000 |
| 7 | -6.599000000 | 4.029000000 | 2.281000000 |
| 6 | -5.880000000 | 3.917000000 | 3.441000000 |

| | | | |
|---|---|---|---|
| 7 | -4.583000000 | 3.958000000 | 3.284000000 |
| 6 | -4.440000000 | 4.039000000 | 1.894000000 |
| 6 | -3.282000000 | 4.113000000 | 1.053000000 |
| 8 | -2.110000000 | 4.157000000 | 1.389000000 |
| 7 | -3.561000000 | 4.145000000 | -0.297000000 |
| 6 | -4.814000000 | 4.121000000 | -0.799000000 |
| 7 | -4.935000000 | 4.102000000 | -2.098000000 |
| 7 | -5.917000000 | 4.064000000 | -0.061000000 |
| 6 | -5.668000000 | 4.030000000 | 1.278000000 |
| 1 | -6.350000000 | 3.826000000 | 4.406000000 |
| 1 | -2.760000000 | 4.211000000 | -0.899000000 |
| 1 | -4.131000000 | 4.079000000 | -2.723000000 |
| 1 | -5.878000000 | 4.015000000 | -2.429000000 |
| 7 | 3.844000000 | 8.037000000 | -3.769000000 |
| 6 | 3.684000000 | 7.865000000 | -2.395000000 |
| 8 | 4.609000000 | 7.870000000 | -1.604000000 |
| 7 | 2.409000000 | 7.692000000 | -1.916000000 |
| 6 | 1.271000000 | 7.667000000 | -2.690000000 |
| 8 | 0.184000000 | 7.569000000 | -2.130000000 |
| 6 | 1.502000000 | 7.785000000 | -4.134000000 |
| 6 | 0.361000000 | 7.655000000 | -5.135000000 |
| 6 | 2.760000000 | 7.978000000 | -4.613000000 |
| 1 | 2.298000000 | 7.618000000 | -0.913000000 |
| 1 | -0.474000000 | 7.115000000 | -4.687000000 |
| 1 | 0.018000000 | 8.645000000 | -5.435000000 |
| 1 | 0.679000000 | 7.102000000 | -6.021000000 |
| 1 | 2.931000000 | 8.095000000 | -5.675000000 |
| 7 | 6.236000000 | 4.772000000 | -1.533000000 |
| 6 | 5.520000000 | 4.484000000 | -2.664000000 |
| 7 | 4.227000000 | 4.439000000 | -2.493000000 |
| 6 | 4.092000000 | 4.606000000 | -1.111000000 |
| 6 | 2.941000000 | 4.618000000 | -0.259000000 |
| 8 | 1.765000000 | 4.581000000 | -0.585000000 |
| 7 | 3.232000000 | 4.687000000 | 1.087000000 |
| 6 | 4.488000000 | 4.781000000 | 1.576000000 |
| 7 | 4.615000000 | 4.809000000 | 2.875000000 |
| 7 | 5.583000000 | 4.819000000 | 0.824000000 |
| 6 | 5.324000000 | 4.729000000 | -0.513000000 |
| 1 | 5.991000000 | 4.334000000 | -3.619000000 |
| 1 | 2.436000000 | 4.729000000 | 1.697000000 |
| 1 | 3.822000000 | 4.696000000 | 3.505000000 |
| 1 | 5.564000000 | 4.816000000 | 3.199000000 |
| 7 | 3.822000000 | 8.617000000 | 4.716000000 |
| 6 | 2.468000000 | 8.347000000 | 4.523000000 |
| 8 | 1.632000000 | 8.425000000 | 5.408000000 |
| 7 | 2.072000000 | 7.993000000 | 3.254000000 |
| 6 | 2.897000000 | 7.901000000 | 2.155000000 |
| 8 | 2.402000000 | 7.657000000 | 1.058000000 |
| 6 | 4.319000000 | 8.127000000 | 2.440000000 |
| 6 | 5.385000000 | 7.954000000 | 1.369000000 |
| 6 | 4.722000000 | 8.474000000 | 3.689000000 |
| 1 | 1.087000000 | 7.803000000 | 3.118000000 |
| 1 | 6.292000000 | 7.503000000 | 1.776000000 |
| 1 | 5.010000000 | 7.285000000 | 0.602000000 |
| 1 | 5.619000000 | 8.911000000 | 0.905000000 |

| | | | |
|---|---|---|---|
| 1 | 5.769000000 | 8.635000000 | 3.907000000 |
| 7 | 1.726000000 | 4.504000000 | 6.886000000 |
| 6 | 2.886000000 | 4.457000000 | 6.160000000 |
| 7 | 2.719000000 | 4.473000000 | 4.864000000 |
| 6 | 1.326000000 | 4.459000000 | 4.729000000 |
| 6 | 0.475000000 | 4.450000000 | 3.577000000 |
| 8 | 0.798000000 | 4.501000000 | 2.401000000 |
| 7 | -0.872000000 | 4.381000000 | 3.864000000 |
| 6 | -1.365000000 | 4.345000000 | 5.121000000 |
| 7 | -2.658000000 | 4.231000000 | 5.247000000 |
| 7 | -0.618000000 | 4.363000000 | 6.220000000 |
| 6 | 0.719000000 | 4.424000000 | 5.962000000 |
| 1 | 3.854000000 | 4.427000000 | 6.630000000 |
| 1 | -1.480000000 | 4.383000000 | 3.065000000 |
| 1 | -3.279000000 | 4.147000000 | 4.444000000 |
| 1 | -2.980000000 | 4.139000000 | 6.193000000 |
| 1 | 4.755538610 | 8.204852670 | -4.144396920 |
| 1 | -4.801757190 | 8.672096160 | -4.486270110 |
| 1 | -5.356546720 | 8.585729680 | 4.819191540 |
| 1 | 4.141918210 | 8.918749930 | 5.614108750 |
| 1 | 1.634053570 | 4.580418560 | 7.878827310 |
| 1 | -7.592318580 | 4.095799400 | 2.186893460 |
| 1 | -2.016526530 | 4.485266420 | -7.082946590 |
| 1 | 7.213782620 | 4.970782260 | -1.466466160 |
| 19 | -0.127023410 | 6.015675900 | 0.360834330 |

**K@UG$^{T+}$:**

| | | | |
|---|---|---|---|
| 7 | 1.535814000 | -6.421984000 | -0.775097000 |
| 6 | 0.166905000 | -6.336826000 | -0.854376000 |
| 7 | -0.277175000 | -5.178721000 | -1.257984000 |
| 6 | 0.881719000 | -4.418754000 | -1.427333000 |
| 6 | 1.056680000 | -3.036712000 | -1.780387000 |
| 8 | 0.194088000 | -2.192503000 | -2.014071000 |
| 7 | 2.378048000 | -2.633876000 | -1.810229000 |
| 6 | 3.413263000 | -3.461786000 | -1.514115000 |
| 7 | 4.614260000 | -2.952069000 | -1.478832000 |
| 7 | 3.295287000 | -4.744191000 | -1.171760000 |
| 6 | 1.996641000 | -5.173360000 | -1.141343000 |
| 1 | -0.489067000 | -7.163117000 | -0.618506000 |
| 1 | 2.554003000 | -1.663845000 | -2.022071000 |
| 1 | 4.768203000 | -1.966760000 | -1.663766000 |
| 1 | 5.381189000 | -3.512138000 | -1.136144000 |
| 7 | -1.910299000 | -5.190071000 | 2.561195000 |
| 6 | -2.458859000 | -3.943170000 | 2.253525000 |
| 8 | -3.643765000 | -3.656111000 | 2.437914000 |
| 7 | -1.596971000 | -2.994175000 | 1.755650000 |
| 6 | -0.245619000 | -3.149761000 | 1.593903000 |
| 8 | 0.416245000 | -2.239824000 | 1.107166000 |
| 6 | 0.274422000 | -4.422809000 | 2.041559000 |
| 6 | -0.549991000 | -5.396540000 | 2.511852000 |
| 1 | -1.977796000 | -2.089384000 | 1.530043000 |
| 1 | 1.337105000 | -4.588880000 | 1.987545000 |
| 1 | -0.155172000 | -6.346436000 | 2.856205000 |
| 7 | -6.629799000 | -1.573345000 | -1.293373000 |
| 6 | -6.596343000 | -0.201466000 | -1.266298000 |

| | | | |
|---|---|---|---|
| 7 | -5.402537000 | 0.306203000 | -1.437090000 |
| 6 | -4.579114000 | -0.818339000 | -1.573457000 |
| 6 | -3.155562000 | -0.949628000 | -1.752110000 |
| 8 | -2.305231000 | -0.063357000 | -1.827618000 |
| 7 | -2.723961000 | -2.259736000 | -1.828452000 |
| 6 | -3.564182000 | -3.324687000 | -1.752556000 |
| 7 | -3.047971000 | -4.514544000 | -1.828925000 |
| 7 | -4.879581000 | -3.248849000 | -1.580149000 |
| 6 | -5.330911000 | -1.966047000 | -1.494631000 |
| 1 | -7.484680000 | 0.400554000 | -1.125163000 |
| 1 | -1.742333000 | -2.412792000 | -1.992958000 |
| 1 | -2.051117000 | -4.665547000 | -1.841994000 |
| 1 | -3.699889000 | -5.293360000 | -1.850347000 |
| 7 | -5.740783000 | 1.574466000 | 2.255667000 |
| 6 | -4.574556000 | 2.285539000 | 1.933143000 |
| 8 | -4.497525000 | 3.513453000 | 1.923676000 |
| 7 | -3.463117000 | 1.547226000 | 1.588324000 |
| 6 | -3.412693000 | 0.182904000 | 1.484875000 |
| 8 | -2.360345000 | -0.360588000 | 1.160413000 |
| 6 | -4.660013000 | -0.490276000 | 1.780232000 |
| 6 | -5.769225000 | 0.198985000 | 2.161191000 |
| 1 | -2.597300000 | 2.039322000 | 1.461649000 |
| 1 | -4.694433000 | -1.564001000 | 1.704454000 |
| 1 | -6.683448000 | -0.327871000 | 2.417441000 |
| 7 | -1.531289000 | 6.629692000 | -0.815222000 |
| 6 | -0.157486000 | 6.606492000 | -0.864081000 |
| 7 | 0.350437000 | 5.436398000 | -1.151334000 |
| 6 | -0.774854000 | 4.614657000 | -1.276121000 |
| 6 | -0.896849000 | 3.207500000 | -1.538295000 |
| 8 | 0.000914000 | 2.382038000 | -1.700157000 |
| 7 | -2.203876000 | 2.760377000 | -1.583253000 |
| 6 | -3.274975000 | 3.563966000 | -1.359601000 |
| 7 | -4.467766000 | 3.039498000 | -1.412091000 |
| 7 | -3.207665000 | 4.866798000 | -1.100209000 |
| 6 | -1.925850000 | 5.339540000 | -1.071804000 |
| 1 | 0.449542000 | 7.485917000 | -0.689255000 |
| 1 | -2.351018000 | 1.784645000 | -1.795292000 |
| 1 | -4.606911000 | 2.050257000 | -1.584906000 |
| 1 | -5.264303000 | 3.626673000 | -1.234031000 |
| 7 | 1.765048000 | 5.260169000 | 2.343567000 |
| 6 | 2.369024000 | 4.036312000 | 2.024716000 |
| 8 | 3.587012000 | 3.837552000 | 2.063616000 |
| 7 | 1.527514000 | 3.004394000 | 1.669618000 |
| 6 | 0.154554000 | 3.051952000 | 1.661556000 |
| 8 | -0.492983000 | 2.048348000 | 1.371096000 |
| 6 | -0.406134000 | 4.329453000 | 2.030850000 |
| 6 | 0.390931000 | 5.381311000 | 2.358000000 |
| 1 | 1.948128000 | 2.101923000 | 1.512710000 |
| 1 | -1.482234000 | 4.429169000 | 2.070200000 |
| 1 | -0.049764000 | 6.320449000 | 2.665859000 |
| 7 | 6.729477000 | 1.589855000 | -0.840740000 |
| 6 | 6.646429000 | 0.234117000 | -1.043685000 |
| 7 | 5.460248000 | -0.189742000 | -1.374260000 |
| 6 | 4.687482000 | 0.973117000 | -1.390948000 |
| 6 | 3.281410000 | 1.156572000 | -1.623598000 |

| | | | |
|---|---|---|---|
| 8 | 2.423423000 | 0.297726000 | -1.831263000 |
| 7 | 2.884583000 | 2.478545000 | -1.576237000 |
| 6 | 3.731683000 | 3.508058000 | -1.306959000 |
| 7 | 3.212745000 | 4.706798000 | -1.251652000 |
| 7 | 5.039181000 | 3.380968000 | -1.056953000 |
| 6 | 5.464141000 | 2.077760000 | -1.107972000 |
| 1 | 7.496718000 | -0.425781000 | -0.935348000 |
| 1 | 1.906759000 | 2.660252000 | -1.738553000 |
| 1 | 2.218350000 | 4.855481000 | -1.381122000 |
| 1 | 3.796467000 | 5.495374000 | -1.020635000 |
| 7 | 5.402994000 | -2.200293000 | 2.074400000 |
| 6 | 4.117206000 | -2.683201000 | 1.803605000 |
| 8 | 3.819532000 | -3.880731000 | 1.817379000 |
| 7 | 3.143242000 | -1.746099000 | 1.536257000 |
| 6 | 3.306628000 | -0.385776000 | 1.551984000 |
| 8 | 2.366215000 | 0.341107000 | 1.243099000 |
| 6 | 4.627103000 | 0.051432000 | 1.945337000 |
| 6 | 5.623253000 | -0.843354000 | 2.199077000 |
| 1 | 2.207749000 | -2.074645000 | 1.358317000 |
| 1 | 4.803826000 | 1.113109000 | 2.040906000 |
| 1 | 6.597877000 | -0.494867000 | 2.514291000 |
| 1 | -6.556641000 | 2.068596000 | 2.556015000 |
| 1 | -2.118308000 | 7.418228000 | -0.631865000 |
| 1 | 2.334947000 | 6.052198000 | 2.562435000 |
| 1 | 7.534134000 | 2.112755000 | -0.559489000 |
| 1 | 6.164289000 | -2.840396000 | 2.177837000 |
| 1 | 2.080910000 | -7.217124000 | -0.509350000 |
| 1 | -2.514118000 | -5.943654000 | 2.821032000 |
| 1 | -7.431367000 | -2.161993000 | -1.188571000 |
| 19 | 0.177836000 | -0.178847000 | -0.211992000 |
